# Optimized genetic circuitry and reporters for sensitive whole-cell arsenic biosensors: advancing environmental monitoring

**DOI:** 10.1128/aem.00601-25

**Published:** 2025-07-10

**Authors:** Yan Guo, Ming-qi Liu, Xue-qin Yang, Ying-yan Guo, Chang-ye Hui

**Affiliations:** 1 National Key Clinical Specialty of Occupational Diseases, Shenzhen Prevention and Treatment Center for Occupational Diseases 574722 , Shenzhen, China; 2 School of Public Health, Guangdong Medical University 12453, Dongguan, China; 3 Pathology and Toxicology Institute, Shenzhen Prevention and Treatment Center for Occupational Diseases 574722 , Shenzhen, China; Kyoto University, Kyoto, Japan

**Keywords:** arsenic pollution, whole-cell biosensors, genetic circuits, indigoidine pigment, ArsR regulatory system, environmental monitoring

## Abstract

The ubiquitous presence of arsenic pollution poses a significant threat to both ecosystem integrity and human health, necessitating the development of sensitive methods for arsenic detection. This study presents the development of innovative whole-cell biosensors that leverage the ArsR regulatory system within both naturally coupled and non-coupled genetic circuits, specifically optimized for detecting the highly toxic arsenic species (As(III)). These biosensors incorporate an indigoidine pigment as a reporting system and utilize the glycerol facilitator protein (GlpF) to enhance arsenic transport, offering a significant advantage over conventional detection methods by streamlining the detection process and eliminating the requirement for specialized instrumentation. Our findings reveal that the indigoidine-based biosensor, TOP10/pnK12-ABS-ind, in particular, exhibits an extensive linear detection range of 0.039 to 20 μM across various water matrices, effectively adhering to and exceeding the arsenic detection guidelines set by the World Health Organization and Chinese national standards. This research advances arsenic biosensing technology by developing a practical, cost-effective detection solution for arsenic in various aquatic settings. Compared to our previous work, this study demonstrates significant improvements in detection range and sensitivity while highlighting the importance of tailored genetic circuit design based on the reporter’s choice.

**IMPORTANCE:**

Arsenic pollution poses a significant threat to global ecosystems and human health, with millions of people at risk of exposure through contaminated water sources. Detecting arsenic, especially in its highly toxic form (As(III)), is crucial for environmental monitoring and public health protection. However, conventional detection methods often require costly equipment and specialized expertise, limiting their feasibility in resource-limited regions. Our study addresses this challenge by developing whole-cell biosensors that leverage natural genetic circuits and a novel indigoidine pigment reporter. These biosensors offer a practical, cost-effective, and portable solution for arsenic detection, streamlining the process and eliminating the need for complex instrumentation. By enabling real-time monitoring and on-site analysis, our biosensors have the potential to significantly enhance environmental monitoring capabilities, facilitate timely remediation efforts, and safeguard public health in areas affected by arsenic contamination.

## INTRODUCTION

Arsenic pollution threatens aquatic ecosystems, human health, and the environment globally. This toxic metalloid, occurring naturally in aquatic and terrestrial environments, has been identified as one of the most prevalent environmental toxins, with severe public health implications (1). Recent data indicates that arsenic contamination affects over 115 countries worldwide, with approximately 94 to 220 million people at risk of high concentrations of arsenic in groundwater, primarily in Asia (2, 3). The contamination of water resources, particularly in regions with sulfide mineralization and smelting activities, has led to arsenic concentrations ranging from 1.3 to 66.7 nM, with extreme values reaching 18.5 and 78.1 nM near hydrothermal systems (4). The presence of arsenic in water bodies directly affects aquatic organisms through inhalation and ingestion and indirectly impacts human health by contaminating drinking water and aquatic food products (4, 5). Therefore, understanding arsenic’s environmental behavior and biological fate is crucial for regulating the environmental risks of arsenic pollution and developing effective remediation strategies.

The intricacies of arsenic speciation and its profound implications for environmental and human health underscore the critical need to develop effective arsenic detection methodologies. Conventional analytical techniques, such as inductively coupled plasma mass spectrometry and inductively coupled plasma atomic emission spectroscopy, are recognized for their high sensitivity and specificity. However, these methods are often constrained by the need for costly equipment and specialized technical knowledge, rendering them less feasible in regions where arsenic contamination is most widespread. This constraint is especially salient in the context of detecting the highly toxic form of arsenic, arsenite (As(III)), which calls for analytical methods that are both low threshold and highly sensitive (6).

ArsR is a transcriptional regulator that senses As(III) by binding to specific DNA sequences (ArsR-binding sites [ABS]) in the promoter region of resistance genes (7). Upon As(III) binding, ArsR undergoes a conformational change that reduces its affinity for DNA, thereby allowing the expression of downstream resistance genes (8). This mechanism forms the basis for As(III) whole-cell biosensor design. The emergence of whole-cell biosensors offers a novel and alternative strategy that addresses these challenges. These biosensors harness the innate metabolic pathways of microorganisms to detect and respond to arsenic, presenting a biological solution to a chemical challenge. They hold particular promise for detecting As(III) due to their potential for heightened sensitivity and specificity and the capability to engineer them for tailored environmental conditions (7). Furthermore, whole-cell biosensors can provide real-time monitoring and are well-suited for field deployment, a precious feature in areas with limited access to laboratory facilities.

The utilization of these biosensors for detecting As(III) is of particular significance as it facilitates the assessment of bioavailable arsenic, which is more pertinent for gauging environmental impacts and health risks. This approach contrasts with traditional methods that may fail to differentiate between various arsenic species, potentially leading to underestimating the risks associated with the more toxic forms (9). Consequently, developing and optimizing whole-cell biosensors for arsenic detection marks a significant stride forward in environmental monitoring and public health safeguarding.

Our previous review (7) comprehensively analyzed the design of arsenic biosensors based on the *ars* operon template, highlighting the importance of genetic circuit design and reporter selection. We demonstrated that using pigment biosynthesis as a reporting system amplifies signals through enzymatic cascades and allows for naked-eye detection, thereby simplifying the detection process. Subsequently, we developed a visual arsenic biosensor using deoxyviolacein (DV) as the pigment reporter (10). This biosensor achieved a detection range of 0.071 to 1.125 µM for As(III), covering relevant environmental limits. However, several questions remained unresolved: (i) the lipophilic nature of DV necessitates extraction, complicating the detection process; (ii) the narrow quantitative range of the DV-based sensor may be related to the choice of reporter and whether it can be expanded; and (iii) whether the successful strategies for *ars* operon template selection, genetic circuit design, and As(III) transport protein GlpF introduction can be generalized to other reporters.

**Fig 1:**
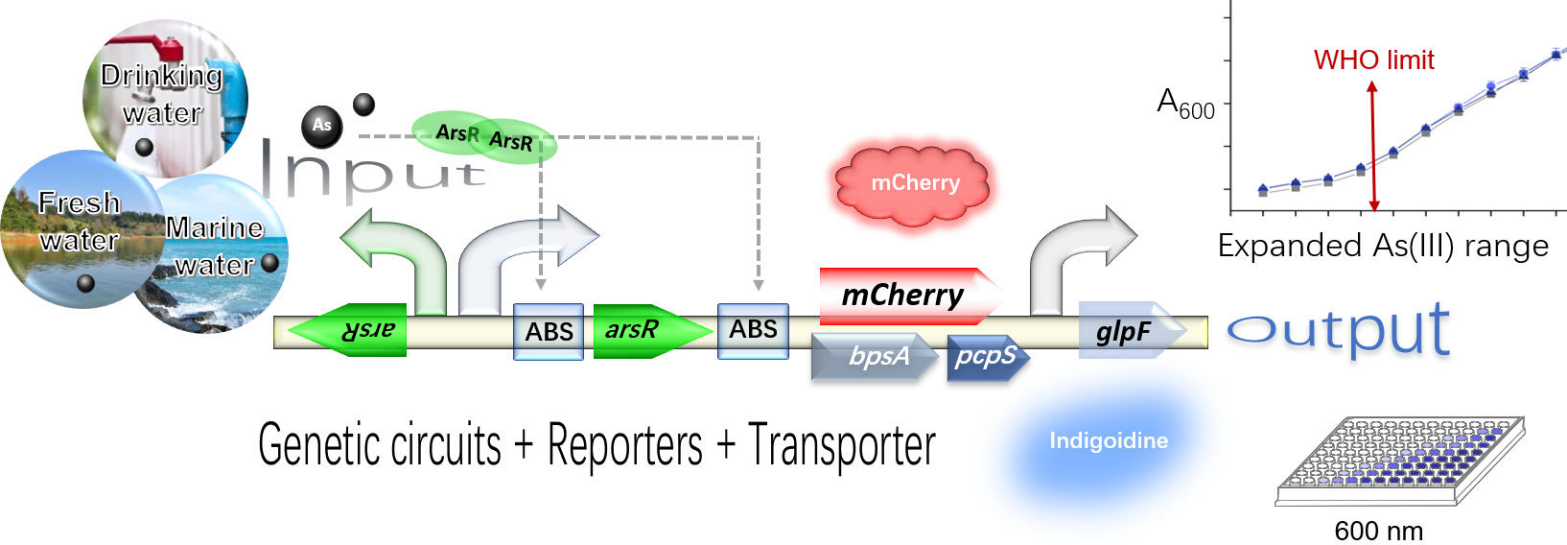
Schematic of As(III) biosensor development strategy.

To address these questions, we conducted the present study (as shown in Fig. 1). We retained the *E. coli* K12 chromosomal *ars* operon template, which was previously optimized. We selected mCherry a widely used fluorescent reporter, and indigoidine, a water-soluble pigment, as representative reporters. The significance of our study lies in two aspects. Practically, the optimized indigoidine-based sensor (TOP10/pnK12-ABS-ind) achieved a significantly broader detection range of 0.039 to 20 µM for As(III) in environmental waters while maintaining a detection limit comparable to that of our previous sensor. The water-soluble nature of indigoidine also simplifies the detection process. Theoretically, our findings emphasize that optimization strategies for one reporter cannot be indiscriminately applied to others. Existing studies primarily focus on optimizing gene circuits (11–13). However, a comprehensive comparison of different reporting systems is necessary. Our study also underscores the importance of comprehensively comparing the performance of different reporting systems. Furthermore, our results suggest that the selectivity of biosensors is entirely dependent on the metal-responsive transcriptional regulators rather than the choice of reporter. Our study advances the field of arsenic biosensing by developing a practical, cost-effective, and broadly applicable sensor for environmental monitoring. It also provides valuable insights into optimizing whole-cell biosensors, emphasizing the need for tailored genetic circuit design based on the choice of reporter.

## RESULTS

### Performance examination of fluorescence-based biosensors using naturally coupled circuit

**Fig 2:**
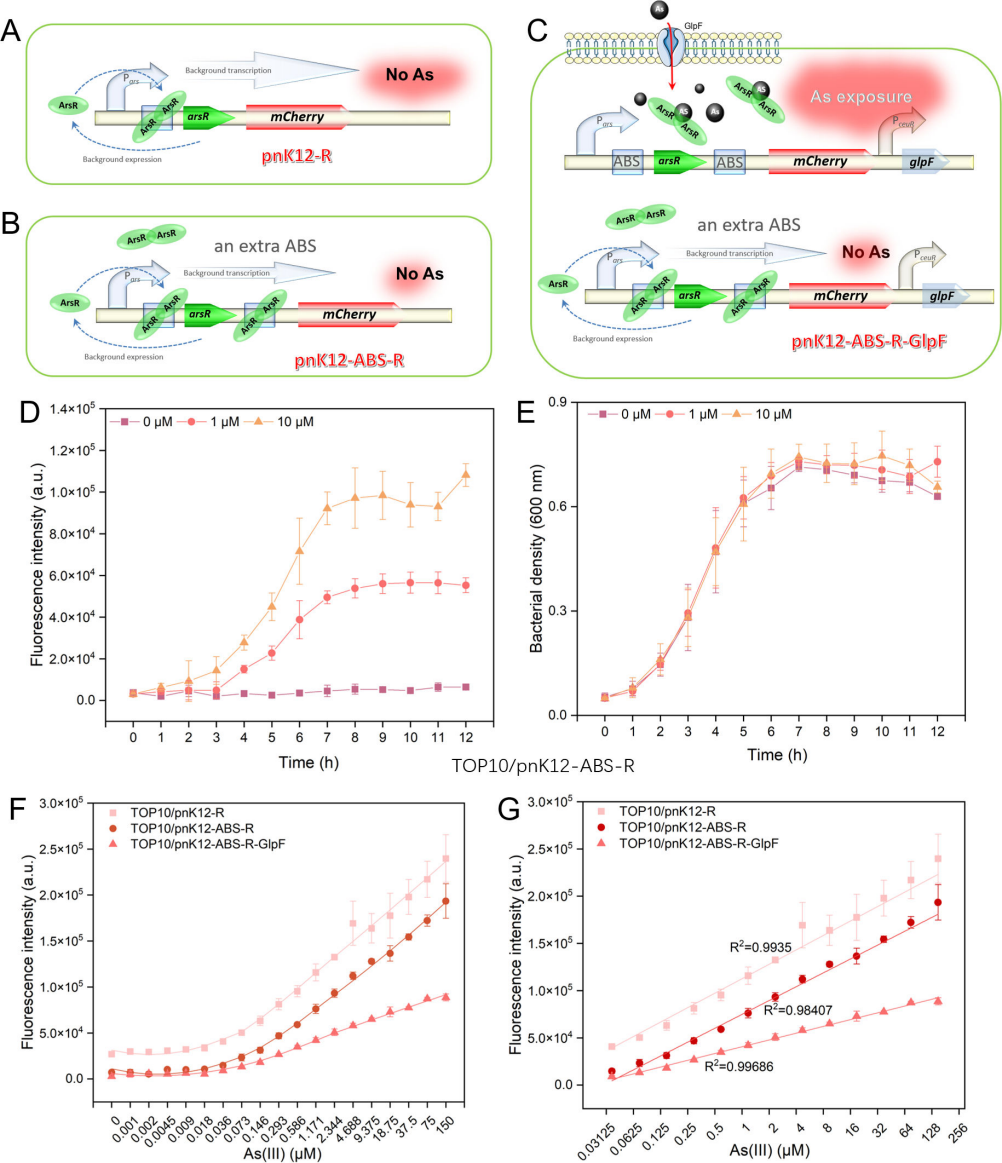
Biosensor performance evaluation based on fluorescent signaling and coupled genetic circuits. (**A**) Schematic representation of the arsenic biosensing genetic construct (pnK12-R), engineered based on an innately coupled genetic circuit. (**B**) The schematic detailing the genetic modification to introduce an ABS proximal to the mCherry fluorescent protein gene within the construct depicted in (**A**) resulted in the new biosensor pnK12-ABS-R. (**C**) Schematic illustration of the genetic architecture of pnK12-ABS-R-GlpF, showcasing the incorporation of the GlpF under the regulation of the P*ceuR* constitutive promoter downstream of the *vioABCE* gene cluster as described in (**B**). (**D**) Chronologically assessed the dose–response profile for the TOP10/pnK12-ABS-R biosensor alongside the concurrent bacterial growth curve (**E**). (**F**) Comparative dose–response profiles of the biosensors TOP10/pnK12-R, TOP10/pnK12-ABS-R, and TOP10/pnK12-ABS-R-GlpF. (**G**) Graphical regression analysis elucidates the correlation between As(III) concentrations spanning 0.036 to 150 µM and the corresponding fluorescence emission, indicative of biosensor performance.

**Fig 3:**
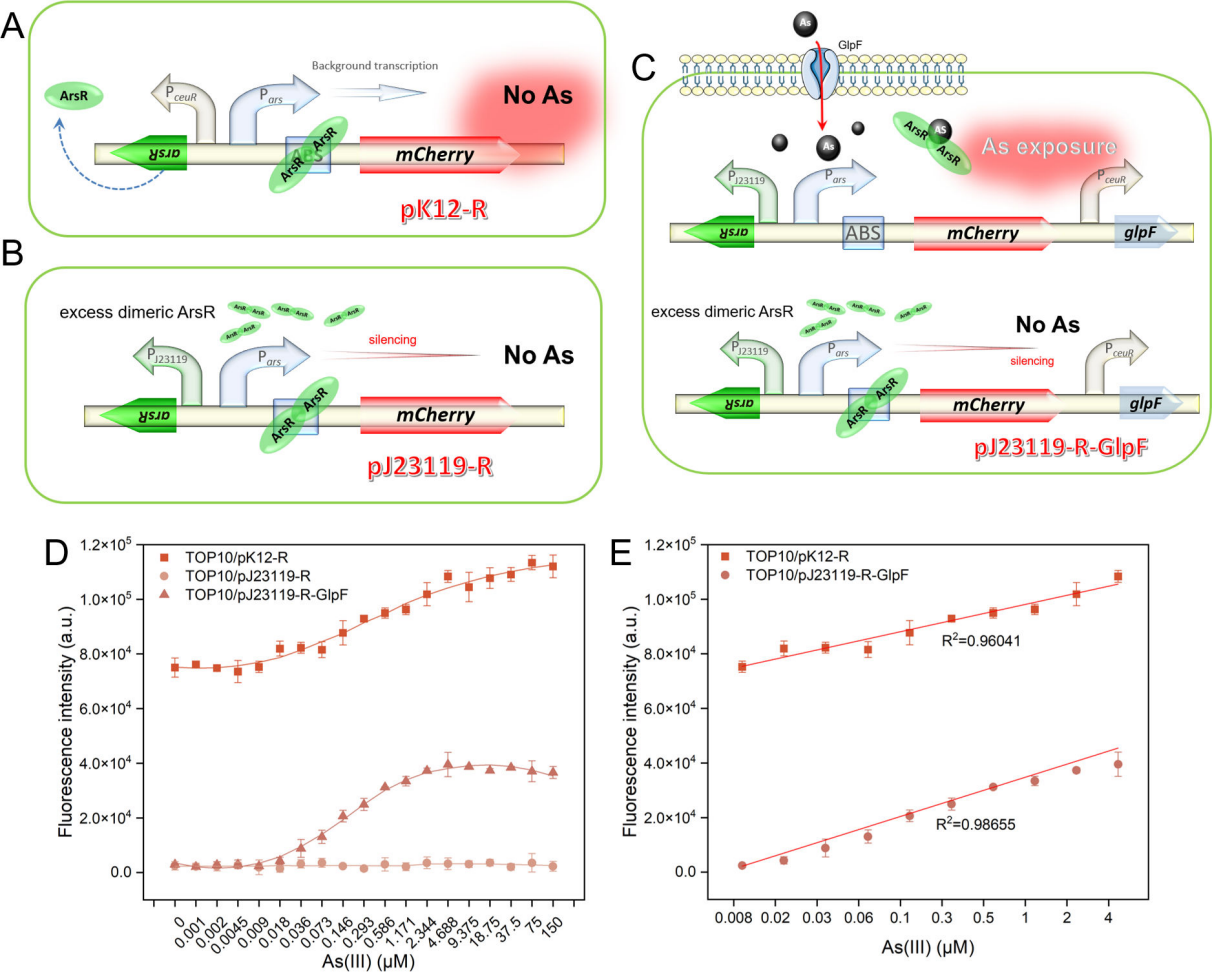
Biosensor performance evaluation based on red fluorescent protein signaling and non-coupled genetic circuits. (**A**) Schematic representation of the arsenic biosensing construct (pK12-R) developed from a non-coupled circuit, where the P*ceuR* constitutive promoter controls the regulator ArsR. (**B**) Schematic illustration of the non-coupled circuit pJ23119-R, in which ArsR is regulated by the strong constitutive promoter PJ23119. (**C**) Schematic diagram depicting the construction of pJ23119-R-GlpF, which incorporates the GlpF under the regulation of the P*ceuR* constitutive promoter, positioned downstream of the *vioABCE* gene cluster as shown in (**B**). (**D**) Dose–response curves for the biosensors TOP10/pK12-R, TOP10/pJ23119-R, and TOP10/pJ23119-R-GlpF. (**E**) Linear regression analysis illustrating the relationship between As(III) concentrations ranging from 0.009 to 4.688 µM and the corresponding red fluorescence values, thereby evaluating the linearity of the biosensors' responses.

Our study leveraged the ArsR regulatory system to develop whole-cell biosensors for arsenic detection. In the initial phase of our study, we conducted preliminary experiments using a fluorescent protein-based reporting system to evaluate the performance of our biosensors. Biosensors based on naturally coupled genetic circuits, including TOP10/pnK12-R (Fig. 2A), TOP10/pnK12-ABS-R (Fig. 2B), and TOP10/pnK12-ABS-R-GlpF (Fig. 2C), as well as those based on non-coupled genetic circuits, namely TOP10/pK12-R (Fig. 3A), TOP10/pJ23119-R (Fig. 3B), and TOP10/pJ23119-R-GlpF (Fig. 3C), were cultured overnight and then subcultured into fresh LB medium at a 1% dilution. These strains were exposed to As(III) concentrations ranging from 0 to 150 µM at 37°C with 250 rpm agitation for four hours. The subsequent changes in red fluorescence intensity were analyzed, as presented in Fig. S1. Notably, biosensors constructed on the naturally coupled genetic circuit, particularly TOP10/pnK12-R and TOP10/pnK12-ABS-R, exhibited superior dose–response relationships, with TOP10/pnK12-ABS-R showing significantly reduced background fluorescence.

Building on these findings, we subsequently focused on the performance of biosensors modified based on naturally coupled circuits, as detailed in Fig. 2A through C. The TOP10/pnK12-ABS-R strain, which demonstrated the most promising results in the preliminary experiments, was subjected to a time–dose–response assessment (Fig. 2D). No background fluorescence was detected in the absence of As(III) induction at 0 µM. Upon exposure to 1 µM and 10 µM As(III), the red fluorescence intensity of the TOP10/pnK12-ABS-R strain increased with extended induction periods, maintaining a distinct dose–response relationship. As depicted in Fig. 2E, there are no significant differences in bacterial density among the three As(III)-induced strains, and the red fluorescence intensity plateaued after seven hours of induction. Consequently, a seven-hour incubation period was selected for subsequent experiments utilizing red fluorescent protein as the reporter.

Lastly, we scrutinized the dose–response relationship of biosensors based on coupled genetic circuits. Figure 2F illustrates that all three biosensors displayed a robust dose–response relationship in detecting As(III), with red fluorescence intensity incrementally rising as the As(III) concentration increased within a nontoxic range. They all demonstrated an excellent linear regression relationship with fluorescence values across a broad concentration range of 0.036–150 μM As(III), as shown in Fig. 2G. The TOP10/pnK12-R strain exhibited higher background fluorescence, while the TOP10/pnK12-ABS-R strain achieved a reduced background but with some compromise in fluorescence response intensity. The TOP10/pnK12-ABS-R-GlpF strain, despite its low background fluorescence, showed a marked decrease in fluorescence intensity in response to As(III). Interestingly, the LODs for all three strains—TOP10/pnK12-R, TOP10/pnK12-ABS-R, and TOP10/pnK12-ABS-R-GlpF—were uniformly determined to be 0.036 µM. The detailed calculation of LOD is described in the Materials and Methods section.

### Performance evaluation of fluorescence-based biosensors using an uncoupled circuit

We investigated the performance of three fluorescent protein-reporting biosensors, each engineered with a distinct non-coupled genetic circuit, as illustrated in Fig. 3A through C. The corresponding dose–response relationships are depicted in Fig. 3D. Among these, the biosensor TOP10/pK12-R showed significantly elevated background fluorescence, whereas TOP10/pJ2319-R and TOP10/pJ23119-R-GlpF exhibited negligible background fluorescence leakage. Interestingly, TOP10/pJ2319-R failed to manifest a dose–response relationship with varying As(III) concentrations, which deviated from our initial expectations. In stark contrast, TOP10/pK12-R and TOP10/pJ23119-R-GlpF exhibited a robust linear response to As(III) concentrations from 0.009 to 4.688 µM (Fig. 3E). The calculated LODs were determined to be 0.293 µM for TOP10/pK12-R and an impressive 0.036 µM for TOP10/pJ23119-R-GlpF. These results emphasize the notable variability in performance among biosensors and highlight the essential role of genetic circuit design in improving the sensitivity and responsiveness of biosensors targeting As(III).

### Performance evaluation of indigoidine-based biosensors

**Fig 4:**
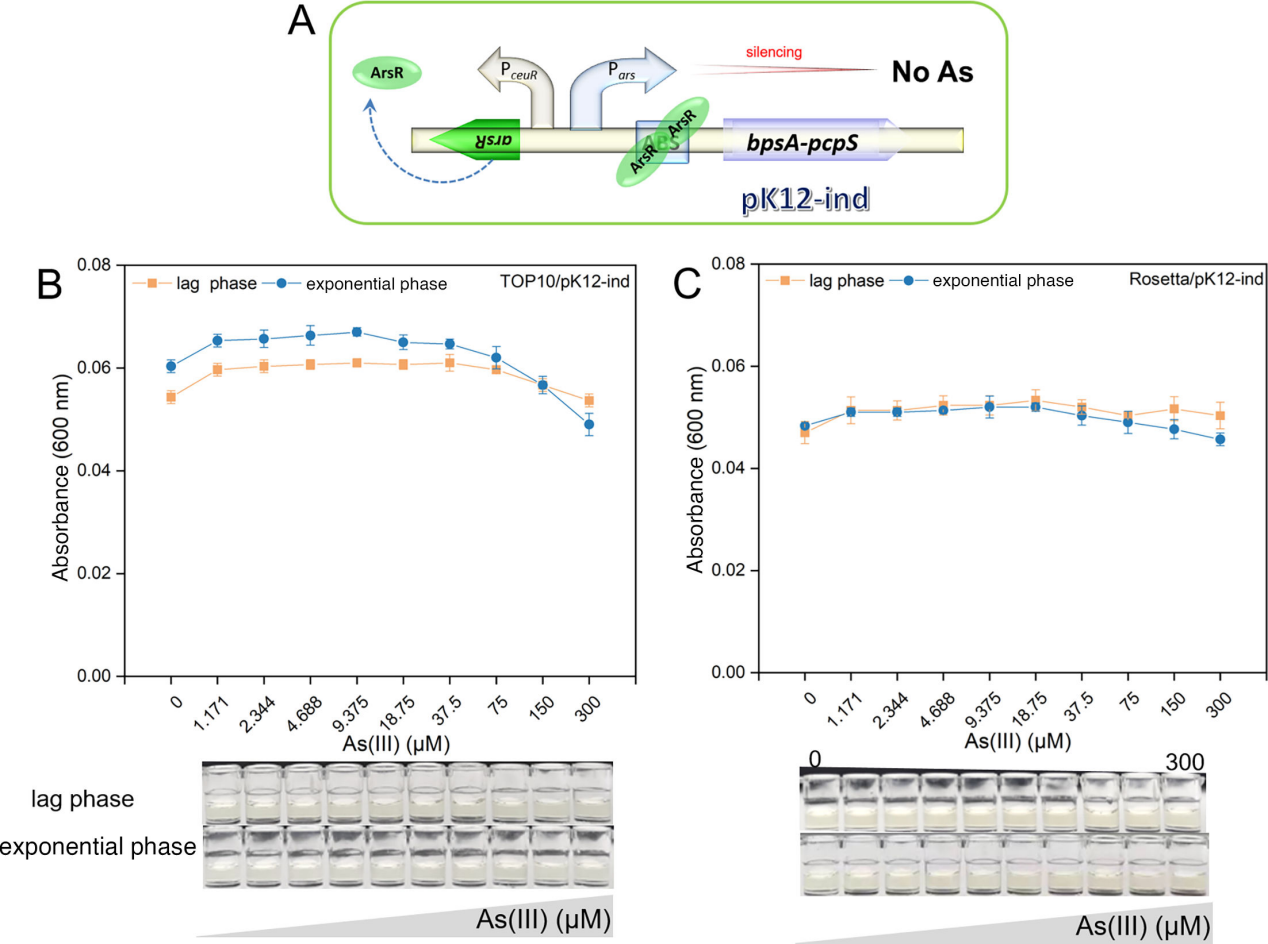
Indigoidine dose–response assessment in non-coupled biosensors. (**A**) Schematic diagram of the arsenic biosensing construct (pK12-ind), based on a non-coupled genetic circuit with the ArsR regulator under the control of the P*ceuR* constitutive promoter. (**B**) Dose–response curves for the recombinant strain TOP10/pK12-ind, illustrating the response to As(III) exposure during the lag and logarithmic growth phases. (**C**) Dose–response curves for the recombinant strain Rosseta(DE3)/pK12-ind, demonstrating the response to As(III) exposure during the lag and logarithmic growth phases, accompanied by representative images of the culture supernatants, which show no significant indigoidine accumulation in any of the treatment groups.

As depicted in Fig. 4A, the architecture of indigoidine-based biosensors pK12-ind (Fig. 4A), constructed on non-coupled genetic circuits, is elaborated. Interestingly, both the cloning host *E. coli* TOP10 (Fig. 4B) and the protein expression host Rosseta(DE3) (Fig. 4C) failed to demonstrate a notable elevation in indigoidine-specific absorbance at 600 nm following exposure to a spectrum of As(III) concentrations (0–300 μM), irrespective of their cellular growth stages, including the lag and logarithmic phases. This absence of a significant absorbance increase at 600 nm suggests a lack of indigoidine synthesis, highlighting the biosensors’ unresponsiveness to As(III) induction.

**Fig 5:**
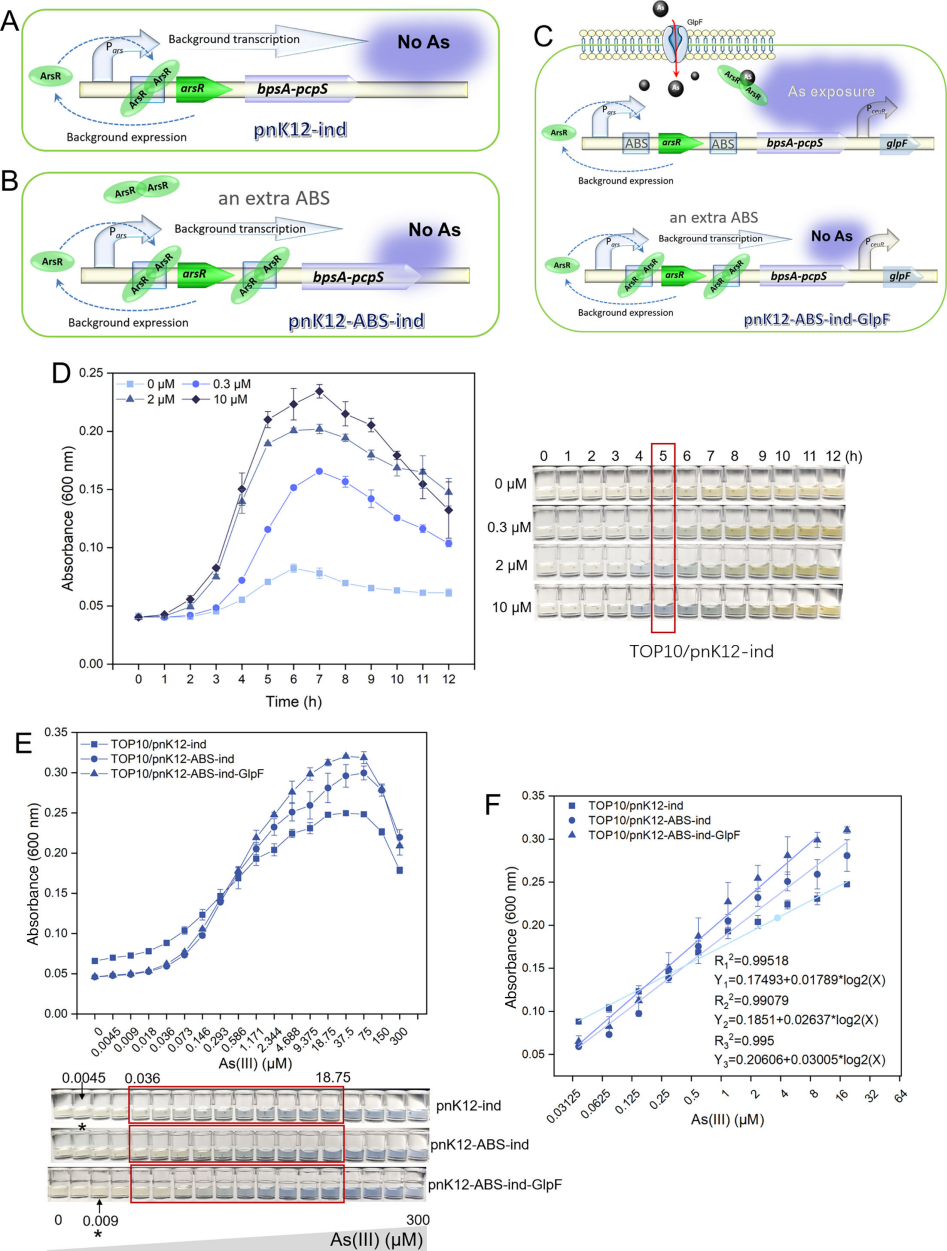
Refinement of indigoidine-based biosensors employing coupled genetic circuits for enhanced arsenic detection. (**A**) Schematic depiction of the arsenic biosensing genetic component (pnK12-ind), engineered within a naturally coupled genetic framework. (**B**) Schematic detail of the pnK12-ABS-ind construct, highlighting the strategic addition of an ABS upstream of the *bpsA-pcpS* gene cluster as outlined in (**A**). (**C**) Schematic representation of the pnK12-ABS-ind-GlpF construct, illustrating the incorporation of the *glpF* gene, under the control of the P*ceuR* constitutive promoter, downstream of the *bpsA-pcpS* gene cluster as detailed in (**B**). (**D**) The comprehensive time–dose response curve for the TOP10/pnK12-ind strain, complemented by the corresponding bacterial growth trajectory and accompanied by representative supernatant images, demonstrates indigoidine production. (**E**) Comparative dose–response curves for the biosensor strains TOP10/pnK12-ind, TOP10/pnK12-ABS-ind, and TOP10/pnK12-ABS-ind-GlpF, with supernatant images at the bottom. An asterisk denotes the LOD, and the linear dynamic range is highlighted within a red square. (**F**) Regression analysis elucidates the correlation between As(III) concentrations spanning 0.036 to 18.75 µM and the indigoidine pigment signal, indicative of the biosensors’ analytical sensitivity and dynamic range. The order of the regression equations corresponds directly to the order presented in the figure captions.

We then concentrated on the performance of biosensors derived from naturally coupled genetic circuits, as illustrated in Fig. 5A through C. The biosensor TOP10/pnK12-ind (Fig. 5A) was subjected to a time–dose–response analysis (Fig. 5D), revealing minimal baseline pigment production at 0 µM As(III) exposure. With increasing concentrations of As(III) at 0.3, 2, and 10 µM, the characteristic absorbance of indigoidine at 600 nm (A_600_ values) escalated progressively over the induction period, sustaining a clear dose–response correlation. A noticeable decrease in A_600_ values after five hours of induction was attributed to the oxidation of indigoidine, shifting its color from blue to yellow (14). Thus, a five-hour incubation period was deemed optimal for biosensing detection.

Subsequently, we scrutinized the dose–response dynamics of the biosensors predicated on coupled genetic circuits. The experimental data (Fig. 5E) demonstrated that all three biosensors displayed a potent dose–response relationship in the detection of As(III), with A_600_ values rising incrementally as As(III) concentrations escalated within a subtoxic range. The deepening blue hue, indicative of As(III) interaction, was also discernible to the naked eye from the photographic images of the colorimetric cups. In comparison with TOP10/pnK12-ind, both TOP10/pnK12-ABS-ind (Fig. 5B) and TOP10/pnK12-ABS-ind-GlpF (Fig. 5C) exhibited diminished background noise and a pronounced elevation in A_600_ values in response to higher As(III) concentrations, with TOP10/pnK12-ABS-ind-GlpF showing the most marked effect. Moreover, all three biosensors manifested an outstanding linear response relationship between As(III) concentrations spanning 0.036 to 18.75 µM and the characteristic absorbance of indigoidine at 600 nm (Fig. 5F). The calculated LODs were 0.045 µM for TOP10/pnK12-ind, 0.045 µM for TOP10/pnK12-ABS-ind, and 0.09 µM for TOP10/pnK12-ABS-ind-GlpF.

### Selectivity assay

Compared to uncoupled genetic circuits, biosensors utilizing fluorescent protein and indigoidine pigment-based reporters demonstrated superior performance within coupled genetic circuits. While the linear response range for As(III) detection was broader in fluorescent protein-based biosensors based on coupled genetic circuits (0.036 to 150 µM), significantly more expansive than that of indigoidine-based biosensors (0.036 to 18.75 µM), the visible color change of indigoidine offers a substantial reduction in the application threshold for visible spectrophotometry. Despite the narrower linear range, it fully satisfies the requirements for environmental monitoring. We selected the indigoidine-based biosensor TOP10/pnK12-ABS-ind for subsequent studies based on a coupled genetic circuit modification.

**Fig 6:**
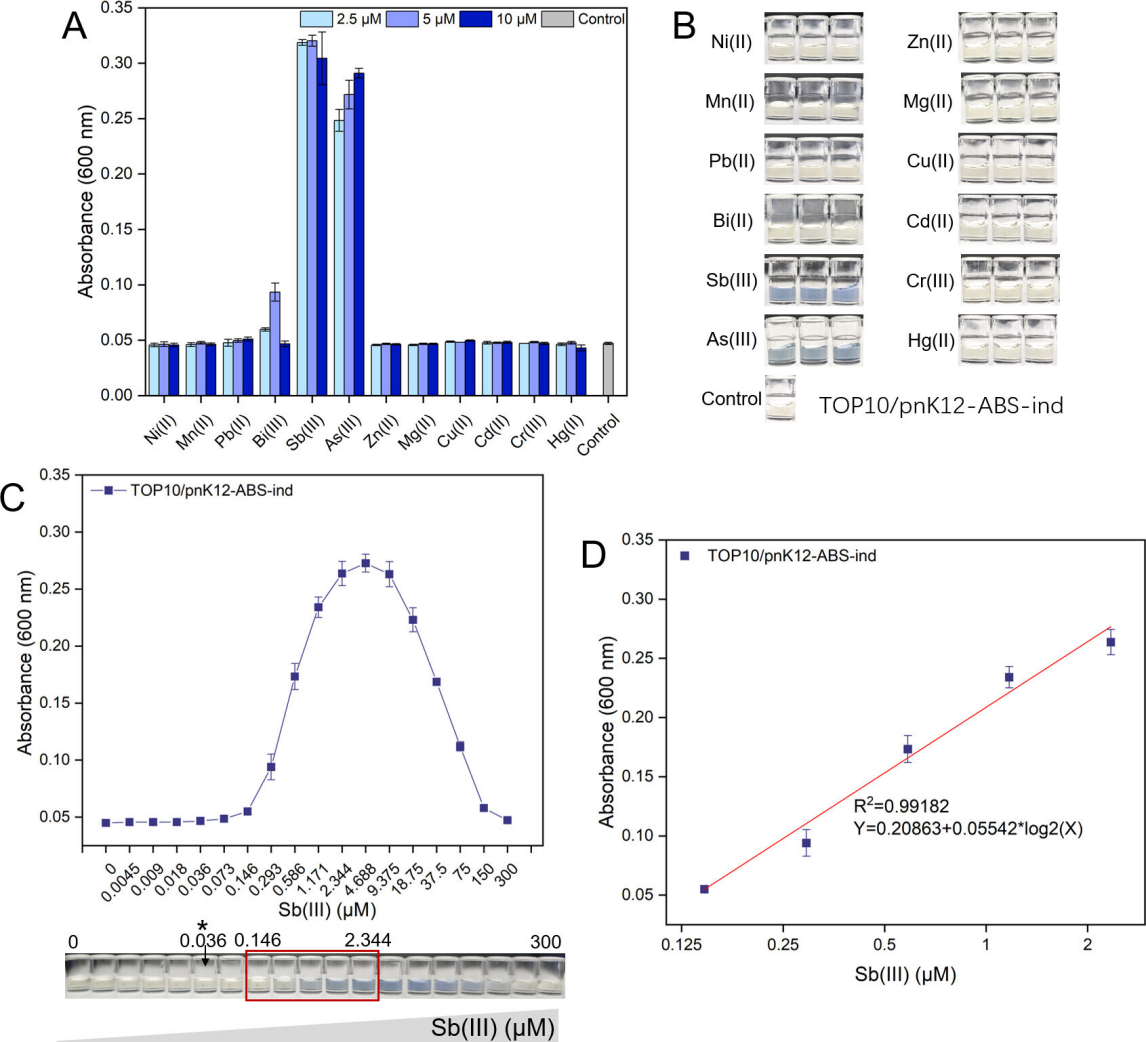
Selective metal response profiling of the biosensor TOP10/pnK12-ABS-ind. (**A**) Graphical representation of the sensor’s pigment-based response to a spectrum of metals, including As(III). (**B**) A series of representative supernatant images from cultures exposed to various metals demonstrate the sensor’s visual response discrimination capabilities. (**C**) Presentation of the dose–response curve for the TOP10/pnK12-ABS-ind biosensor in response to Sb(III), complemented by corresponding supernatant images. The sensor’s linear detection range is indicated by a red square, with an asterisk marking the established detection limit. (**D**) A regression analysis chart detailing the correlation between Sb(III) concentrations within the 0.146 to 2.344 µM range and the indigoidine pigment signal.

We investigated the selective response of TOP10/pnK12-ABS-ind to multiple metals (Fig. 6A). In addition to responding to As(III), it exhibited a strong response to the Group 15 metalloid Sb(III) and a weak response to Bi(III). Notably, the response to As(III) and Sb(III) produced a distinct blue color visible to the naked eye (Fig. 6B). Subsequently, we examined the dose–response relationship of TOP10/pnK12-ABS-ind to Sb(III) (Fig. 6C). The LOD for Sb(III) was calculated to be 0.036 µM, identical to that for As(III). However, the linear response range was significantly narrowed to 0.146–2.344 μM for Sb(III) (Fig. 6D).

### Environmental sample application

**Fig 7:**
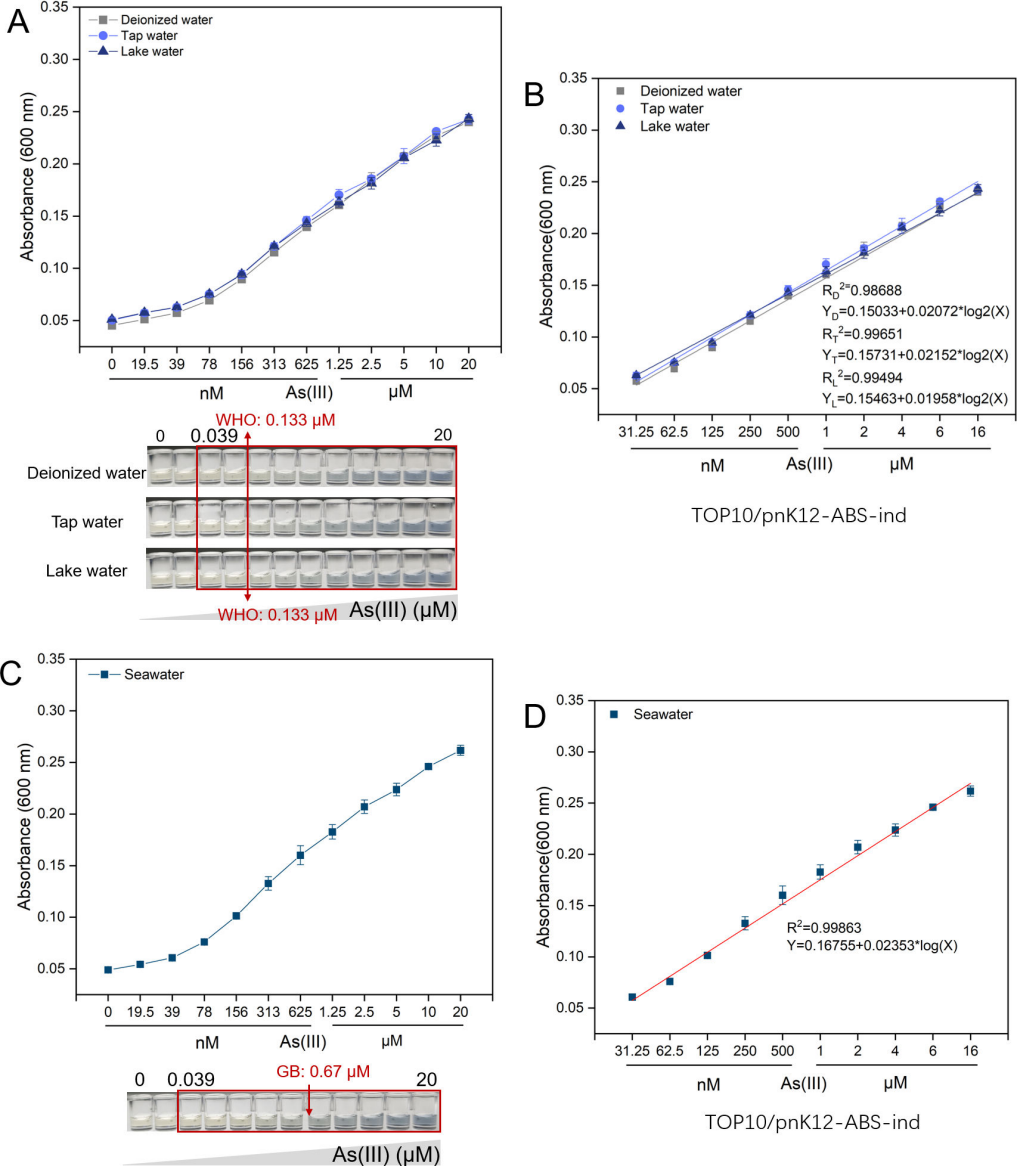
Assessment of the TOP10/pnK12-ABS-ind biosensor for detecting arsenic in spiked environmental water samples. (**A**) The dose–response relationship of the sensor in freshwater samples, encompassing deionized water, tap water, and lake water, is illustrated. Representative images of the culture supernatants are displayed below, with the sensor’s linear detection range marked by a red square and the detection limit indicated by an asterisk. The WHO’s guideline value is also annotated for comparative reference. (**B**) Linear regression analysis delineates the correlation between As(III) concentrations spanning 0.039 to 20 µM and the indigoidine pigment signal, evaluating the sensor’s analytical efficacy in freshwater matrices. (**C**) The dose–response profile of the sensor in seawater samples is presented, with the linear range demarcated by a red square. The GB (National Standard of China) guideline value is highlighted to underscore the sensor’s regulatory relevance. (**D**) Linear regression analysis affirms the sensor’s potential by examining the correlation between As(III) concentrations from 0.039 to 20 µM and the indigoidine pigment signal in seawater samples, reinforcing the sensor’s broader applicability in environmental monitoring.

Our final assessment scrutinized the efficacy of the indigoidine-based biosensor TOP10/pnK12-ABS-ind (Fig. 5B) based on a coupled genetic circuit in detecting As(III) in spiked environmental water samples. Freshwater samples consisted of deionized water, municipal tap water, and lake water from urban parks. Our findings revealed that the biosensor TOP10/pnK12-ABS-ind displayed highly consistent dose–response relationships across these three freshwater matrices (Fig. 7A), closely mirroring the dose–response curve established under controlled laboratory conditions. The linear regression range was determined to be 0.039–20 μM (Fig. 7B), aligning with the quantification range observed in laboratory settings. This range includes the World Health Organization (WHO) drinking water guideline limit of 0.133 µM for arsenic.

Remarkably, the biosensor’s performance in spiked seawater samples paralleled that observed in freshwater and laboratory conditions, as depicted in Fig. 7C. According to the Chinese seawater quality standards (GB 3097-1197), arsenic limits are set at 0.30 µM for Class I, 0.40 µM for Class II, and 0.67 µM for both Class III and IV seawaters. The quantification range for arsenic in seawater, as determined using our developed biosensor, was from 0.039 to 20 μM (Fig. 7D). Accounting for the twofold dilution of seawater in the sensing cultivation system, the adjusted quantification range extended to 0.078–40 μM, which comprehensively encompasses the regulatory limits.

Thus, the biosensor TOP10/pnK12-ABS-ind, developed in this study, is well-suited to meet the monitoring demands for actual environmental water samples, offering a reliable, low-cost, and robust tool for arsenic detection in various aquatic matrices.

## DISCUSSION

Our findings suggest that DV’s optimization strategies do not apply to mCherry and indigoidine. Specifically, in the uncoupled genetic circuit with mCherry, increasing ArsR expression reduced background fluorescence but eliminated the dose-dependent response to As(III). In contrast, indigoidine expression was not detected in the uncoupled circuit. Instead, we found that coupling the genetic circuit and inserting an ArsR-binding site (ABS) upstream of the reporter gene effectively reduced background noise for both mCherry and indigoidine reporters. Interestingly, the introduction of GlpF did not enhance the sensitivity of sensors based on mCherry or indigoidine, contrasting with our previous findings using DV (10).

The strategic selection of genetic circuitry and reporters is crucial for constructing whole-cell biosensors, as it directly influences the sensitivity, specificity, and dynamic range of detection capabilities (11, 12). Our study harnessed the ArsR regulatory system for arsenic detection by engineering biosensors with naturally coupled and non-coupled genetic circuits. Naturally coupled circuits, which emulate the native heavy metal resistance operon, have demonstrated superior dose–response relationships and reduced background noise, essential for precise biosensing (15, 16). In contrast, non-coupled circuits, despite their genetic design flexibility and wide detection range, often contend with high basal expression levels that can mask the detection of low-level target molecules (10, 11). Incorporating an ABS was a deliberate strategy to minimize background noise, enhancing the biosensor’s sensitivity to low arsenic concentrations (10). Previous studies indicate that including ABS effectively lowers basal expression, which is critical for achieving an optimal signal-to-noise ratio (17).

Moreover, our adoption of the indigoidine pigment as a reporter system builds upon the successful utilization of water-insoluble pigments in analogous biosensors (18–21). The water-soluble nature of indigoidine simplifies detection processes. It reduces practical application barriers, offering a significant advantage over traditional fluorescent protein-based reporters that typically necessitate specialized instrumentation for signal interpretation (22). Furthermore, the integration of the glycerol facilitator protein GlpF, recognized for its specificity in As(III) transport (23, 24), was intended to augment the sensitivity of our biosensors. By synergizing these elements, our biosensor design aims to enhance detection capabilities and meet the increasing demand for accessible and efficient arsenic monitoring solutions across diverse environmental settings.

Our comparative analysis of biosensors harnessing red fluorescent protein as the reporting system revealed notable performance disparities, especially when evaluating naturally coupled circuits (Fig. 2) against their non-coupled counterparts (Fig. 3). Regarding background fluorescence, biosensors based on coupled circuits, exemplified by TOP10/pnK12-ABS-R, displayed significantly diminished basal fluorescence intensity compared to those employing non-coupled circuits, such as TOP10/pK12-R, which exhibited heightened background fluorescence. This reduction in basal fluorescence in coupled circuits is credited to the deliberate inclusion of an ABS, which efficiently suppresses leaky expression in the absence of the inducing agent As(III), as previously elucidated (9, 25, 26), thereby augmenting the signal-to-noise ratio and enhancing the biosensors’ overall sensitivity. In terms of detection limits, the non-coupled biosensor TOP10/pJ23119-R-GlpF showcased an exceptional LOD of 0.036 µM for As(III), outperforming the LOD of 0.293 µM observed for the coupled biosensor TOP10/pnK12-ABS-R. This suggests that while non-coupled circuits, augmented with the As(III) transporter GlpF (27), may provide heightened sensitivity at exceedingly low As(III) concentrations, coupled circuits offer a more robust response profile with minimized background interference, which is instrumental for precise quantification in complex environmental matrices. Considering the linear dynamic range, coupled circuit biosensors extended a broader range of 0.036 to 150 µM for As(III) detection, in contrast to the non-coupled biosensor TOP10/pJ23119-R-GlpF, which spanned a narrower range of 0.009 to 4.688 µM. The extended dynamic range of the coupled circuits underscores their capacity to respond to a broader concentration gradient of As(III), rendering them more amenable to applications anticipating variable arsenic concentrations. The juxtaposition of coupled and non-coupled genetic circuits underscores the inherent trade-offs between sensitivity, detection limits, and dynamic range. Although non-coupled circuits may excel in detecting trace levels of arsenic due to their lower LOD, coupled circuits deliver a more balanced performance portfolio characterized by diminished background fluorescence and an expanded linear response range, which confers a distinct advantage for environmental monitoring and detection endeavors.

Our comprehensive analysis of indigoidine-based biosensors, utilizing both non-coupled (Fig. 4) and coupled genetic circuits (Fig. 5), unveiled significant performance disparities. Contrary to our expectations, the non-coupled indigoidine biosensors, as illustrated in Fig. 4, failed to manifest a dose–response relationship. This result was unanticipated and starkly contrasts the coupled biosensors’ performance. The latter, exemplified by the TOP10/pnK12-ABS-ind strain, exhibited a marked increase in indigoidine absorbance at 600 nm in response to As(III) induction, indicative of an enhanced sensitivity attributed to the strategic positioning of an ABS upstream of the pigment-encoding genes. In terms of detection limits, the coupled biosensor TOP10/pnK12-ABS-ind demonstrated a LOD of 0.045 µM for As(III), a sensitivity comparable to that of red fluorescent protein-based biosensors, such as TOP10/pnK12-ABS-R (0.036 µM) and TOP10/pJ23119-R-GlpF (0.036 µM). This equivalence implies that while indigoidine-based biosensors offer a visible response, their sensitivity matches fluorescent biosensors, making them strong candidates for environmental monitoring applications that require quick and cost-effective detection methods. Considering the linear dynamic range, coupled indigoidine-based biosensors such as TOP10/pnK12-ABS-ind covered a range of 0.036 to 18.75 µM for As(III) detection. Although this range is narrower than the red fluorescent protein-based biosensors, which spanned 0.036 to 150 µM, it still encompasses the WHO drinking water guideline limit for arsenic. The narrower range observed in indigoidine-based biosensors may be attributed to pigment production saturation at elevated As(III) concentrations, potentially limiting their application in environments with a broad spectrum of arsenic concentrations. In conclusion, while both red fluorescent protein- and indigoidine-based biosensors have their distinct advantages, the latter offers a visually accessible and cost-effective arsenic detection solution with sensitivity that is comparable to the former. The visual readout afforded by indigoidine-based biosensors simplifies the detection process and minimizes the requirement for specialized equipment, thereby reducing barriers to arsenic detection in settings with limited resources.

While our indigoidine-based biosensor demonstrated a nonspecific response to Sb(III) but within a narrow concentration range, the cross-reactivity with Sb(III) is attributed to the chemical similarities with As(III), a widely known limitation of ArsR-based biosensors (10, 28–31). Although our primary objective was to develop a practical arsenic biosensor for field applications, detecting Sb(III) by our biosensor highlights an important environmental concern that should not be overlooked. Antimony (Sb) is an important strategic mineral resource with increasing applications in the new energy industry, such as photovoltaic cells, lithium battery anode materials, and flame retardant production (32). However, the rapid growth of these industries has led to emerging sources of Sb pollution, posing significant risks to ecosystems and human health (33). Given the increasing prevalence of Sb contamination, the ability of our biosensor to detect Sb(III) could serve as an early warning tool for both As and Sb pollution. While our biosensor does not distinguish between As(III) and Sb(III), detecting either metalloid is valuable for environmental monitoring. The presence of Sb(III) in environmental samples can indicate potential contamination from industrial activities, and its detection by our biosensor can prompt further detailed analysis. Future work can address the selectivity challenge through direct evolution, mutation selection, promoter engineering, and extracellular efflux design of Sb(II) (34–36). It will enhance their applicability in complex environmental matrices and ensure more specific detection of As and other metalloids.

In summary, our indigoidine-based biosensor, TOP10/pnK12-ABS-ind, has demonstrated robust environmental applicability across diverse water matrices, with a linear detection range of 0.039 to 20 µM for As(III) in freshwater and seawater samples. This range effectively encompasses the WHO drinking water guideline limit of 0.133 µM and, accounting for the twofold dilution of the seawater matrix during testing, extends from 0.078 to 40 µM, fully covering the GB standards for arsenic across different seawater classes. These results highlight the biosensor’s potential as a reliable tool for arsenic detection in various aquatic settings. However, it is crucial to acknowledge that complex mixtures of metals and other interfering substances often characterize real-world contaminated environments. Although our biosensor has shown high selectivity for As(III) in the presence of various metals, the potential for interference from other contaminants in highly polluted areas cannot be entirely ruled out. The presence of multiple metal ions and organic compounds in polluted water sources may introduce additional challenges, such as competitive binding or altered bioavailability of arsenic, which could impact the biosensor’s performance. Further validation in more complex environmental samples is necessary to ensure the biosensor’s applicability in practical scenarios. It will involve assessing its performance in the presence of a broader range of contaminants and under varying environmental conditions. Such comprehensive validation will be crucial for addressing potential challenges associated with real-world environmental monitoring and ensuring the biosensor’s reliability and accuracy in detecting arsenic in polluted environments. Future work should also explore strategies to mitigate potential interferences, such as optimizing the biosensor’s design or incorporating additional genetic modules to enhance its selectivity and robustness.

## MATERIALS AND METHODS

### Bacterial strains, reagents, and culture conditions

A comprehensive list of bacterial strains and plasmids employed in this research is detailed in Table 1. Custom synthesis of all oligonucleotide primers and DNA fragments was performed by Sangon Biotech (Shanghai, China). *Escherichia coli* (*E. coli*) TOP10 served as the primary host strain for constructing whole-cell biosensors, except the plasmid pK12-ind, which was introduced into both *E. coli* TOP10 and Rosseta(DE3) strains. Various chemicals, including NaAsO_2_, HgCl_2_, CaCl_2_, MgCl_2_, MnSO_4_, NiSO_4_, CuSO_4_, ZnSO_4_, CdCl_2_, Pb(NO_3_)_2_, C_8_H_4_K_2_O_12_Sb_2_·3H_2_O, BiN_3_O_9_·5H_2_O, and additional reagents were sourced from Sangon Biotech (Shanghai, China). All stock solutions were prepared afresh utilizing analytical-grade chemicals and deionized water.

**TABLE 1:** Bacterial strains and plasmids used in this study

Strains and plasmids	Genotypes or description	Reference
*E. coli* strain		
TOP10	F^-^ Φ80*lac*ZΔM15 Δ*lac*X74 *rec*A1	Tiangen
Rosseta(DE3)	F^-^ *ompT hsdS_B_* (*r_B_*^-^ *m_B_*^-^) *gal dcm* (DE3) *pRARE*	Tiangen
Plasmids		
pET-RFP	pET-21a derivative harboring the red fluorescent protein mCherry encoding sequence.	(37)
pT7lac-ind	pET-21a derivative containing the indigoidine biosynthetic module (a *bpsA* and *pcpS* bicistronic cassette) inserted as a *Nde*I/*Sac*I fragment	(38)
pnK12-DV	The *vioABCE* gene cluster fused downstream of a natural arsenic sensory element from *E. coli* K12 chromosomal *ars* operon.	(10)
pnK12-R	The mCherry reporter fused downstream of the natural arsenic sensory element from *E. coli* K12 chromosomal *ars* operon.	This study
pnK12-ABS-R	The mCherry reporter fused downstream of a modified arsenic sensory element, with an extra ArsR-binding site inserted before the reporter.	This study
pnK12-ABS-R-GlpF	pnK12-ABS-R derivative with the *glpF* fused downstream of the mCherry reporter with the expression of GlpF is controlled by a constitutive P*ceuR* promoter.	This study
pK12-R	The mCherry reporter fused downstream of a redesigned arsenic sensory element with the expression of *arsR* is controlled by a constitutive P*cueR* promoter.	This study
pJ23119-R	The mCherry reporter fused downstream of a redesigned arsenic sensory element with the expression of *arsR* is controlled by a strong constitutive PJ23119 promoter.	This study
pJ23119-R-GlpF	pJ23119-R derivative with the *glpF* fused downstream of the mCherry reporter with the expression of GlpF is controlled by a constitutive P*ceuR* promoter.	This study
pK12-ind	The *bpsA-pcpS* gene cluster fused downstream of a redesigned arsenic sensory element with the expression of *arsR* is controlled by a constitutive P*cueR* promoter.	This study
pnK12-ind	The *bpsA-pcpS* gene cluster fused downstream of the natural arsenic sensory element from *E. coli* K12 chromosomal *ars* operon.	This study
pnK12-ABS-ind	The *bpsA-pcpS* gene cluster fused downstream of a modified arsenic sensory element, with an extra ArsR-binding site inserted before the reporter.	This study
pnK12-ABS-ind-GlpF	pnK12-ABS-ind derivative with the *glpF* fused downstream of the *bpsA-pcpS* gene cluster with the expression of GlpF is controlled by a constitutive P637 promoter	This study

To cultivate engineered bacteria, a Luria-Bertani (LB) medium consisting of 10 g/L tryptone, 5 g/L NaCl, and 5 g/L yeast extract was used, adding 50 mg/L ampicillin as needed. The same LB formulation was augmented with 1.5% agar for solid medium preparations. Unless otherwise noted, all experimental procedures were carried out at a controlled temperature of 37°C and agitation speed of 250 rpm, ensuring standardized conditions for reliable biological assays.

### Construction of biosensing plasmids

The mCherry-encoding gene was PCR amplified from pET-RFP and substituted for the *vioABCE* gene cluster in pnK12-DV to generate the plasmid pnK12-R. Subsequently, an additional ArsR-binding site (ABS) was introduced upstream of the mCherry reporter to generate pnK12-ABS-R. The glycerol facilitator (GlpF) was inserted into pnK12-ABS-R to create pnK12-ABS-R-GlpF under the control of the P*ceuR* promoter. Vectors pK12-R and pJ23119-R were constructed using non-coupled circuits with ArsR, controlled by the constitutive promoter P*ceuR* and PJ23119, respectively. Similarly, GlpF, under the control of promoter P*ceuR*, was inserted into pJ23119-R to generate pJ23119-R-GlpF. The indigoidine biosynthetic module, comprising the *bpsA* and *pcpS* bicistronic unit, was PCR amplified from pT7lac-ind and substituted for the *mcherry* gene in plasmids pnK12-R, pK12-R, pnK12-ABS-R, and pnK12-ABS-R-GlpF to generate plasmids pnK12-ind, pK12-ind, pnK12-ABS-ind, and pnK12-ABS-ind-GlpF, respectively. The DNA sequences of all biosensing elements used in this study are detailed in Table S1.

### Temporal dose–response assessment

The objective of the time–dose response assays was to scrutinize the dynamic reaction of whole-cell biosensors to a gradient of As(III) concentrations over time. Isolated colonies of TOP10/pnK12-ABS-R and TOP10/pnK12-ind were cultured in 1 mL of LB medium and incubated overnight. Subsequently, these cultures were diluted into fresh LB medium at a 1% ratio. The TOP10/pnK12-ABS-R strain was subjected to induction with As(III) concentrations of 0, 1, and 10 µM at a controlled temperature of 37°C and agitation rate of 250 rpm for 12 hours, with hourly measurements of bacterial density and fluorescence intensity. Similarly, the TOP10/pnK12-ind strain underwent induction with As(III) concentrations of 0, 0.3, 2, and 10 µM under identical conditions for 12 hours, with hourly bacterial density and indigoidine absorbance assessments. These meticulous evaluations were essential to chart the biosensors’ sensitivity and response kinetics to As(III) at varying concentrations.

### Dose–response analysis

To evaluate the biosensor’s response to a spectrum of As(III) concentrations, we initiated the process by selecting individual colonies and incubating them overnight in 1 mL of culture medium. These cultures were subsequently inoculated into the fresh medium at a 1% dilution. Biosensors based on red fluorescent protein were initially exposed to an As(III) concentration of 300 µM, followed by a geometric progression of dilutions to achieve concentrations ranging from 300 down to 0 µM As(III). Post a seven-hour incubation at 37°C with agitation at 250 rpm, bacterial density was assessed, and fluorescence intensity was quantified. Biosensors utilizing indigoidine as the reporter were processed identically but exposed to As(III) concentrations from 0 to 150 µM and incubated for five hours before measuring bacterial density and indigoidine absorbance at 600 nm. The dose–response relationship for the TOP10/pnK12-ABS-ind strain in response to Sb(III) was evaluated using a method analogous to As(III) detection.

### Assessment of biosensor specificity

To determine the specificity of the biosensor TOP10/pnK12-ABS-ind, we evaluated its response to a panel of metals. Single colonies were selected and incubated overnight in 1 mL of LB medium. Subsequently, these were inoculated into fresh medium at a 1% dilution. The biosensor was challenged with concentrations of 2.5, 5, and 10 µM for As(III), Cr(III), Cd(II), Hg(II), Mn(II), Mg(II), Pb(II), Cu(II), Sb(III), Bi(III), Zn(II), and Ni(II). After incubation at 37°C with 250 rpm shaking for five hours, the bacterial density and indigoidine absorbance at 600 nm were measured to assess the response to each metal. This comprehensive evaluation is crucial for confirming the selectivity of the biosensor towards the target analyte, As(III), in the presence of other potentially interfering metal ions.

### Assessment of arsenic bioavailability in environmental samples

To ensure the elimination of impurities and bacterial contamination, surface water samples from Xianhu Park in the Luohu District of Shenzhen, China, and seawater from Daya Bay in the Yantian District of Shenzhen, China, were subjected to filtration using a 0.22 µm membrane filter. The recombinant bacteria strain TOP10/pnK12-ABS-ind was activated overnight and then inoculated into a 1 mL culture system at a 1% ratio. A mixture of 10% 10 × LB medium and 90% freshwater samples (deionized water, lake water, and tap water) was prepared for the freshwater biosensing system (39). For the seawater biosensing system, a mixture containing 10% NaCl-free 10 × LB medium, 40% purified water, and 50% seawater samples was prepared (40, 41). Ampicillin at 50 µg/mL concentration was incorporated into the culture systems to exert selective pressure. Single colonies of TOP10/pnK12-ABS-ind were selected and cultured overnight in 1 mL of medium, then inoculated at a 1% ratio into the corresponding biosensing medium. A range of As(III) concentrations from 0 to 20 µM was introduced into the culture systems using a twofold dilution method. After incubation at 37°C and 250 rpm for five hours, bacterial density and indigoidine absorbance at 600 nm were measured to assess arsenic bioavailability.

### Quantification of bacterial density, fluorescence intensity, and indigoidine signal

To quantify bacterial density at 600 nm, 100 µL aliquots of the bacterial culture were transferred into a 96-well plate and assessed using a microplate reader (BioTek Epoch, Winooski, VT, USA). The remaining culture was centrifuged at 12,000 rpm for two minutes. Subsequently, 100 µL of the supernatant was carefully transferred to a 96-well plate, and the absorbance of indigoidine at 600 nm was measured utilizing the same microplate reader. The absorbance of the supernatant represents the indigoidine signal. The bacterial density was calculated by subtracting the absorbance of the supernatant from the total absorbance of the uncentrifuged culture at 600 nm.

The fluorescence intensity was evaluated with a fluorescence spectrophotometer (Thermo Fisher Scientific, USA). A 500 µL culture sample was diluted with 2.5 mL of deionized water to achieve a final dilution factor of six. The fluorescence intensity was quantified using excitation and emission wavelengths of 587 nm and 610 nm, respectively. Each experimental condition was replicated three times to ensure the reliability of our findings.

### Data analysis

The fluorescence signals were quantified using the arbitrary units of fluorescence intensity (a.u.) measured by the instrument. The detection limit (LOD) was ascertained using the formulas: Limit of Blank (LOB) = Mean Blank + 1.645 × Standard Deviation (S.D.) of Blank and LOD for low-concentration samples = LOB + 1.645 × S.D. Data presentation on the x-axis was log2-transformed to reflect metal concentration, and the standard curves were derived from linear regression analysis. To ensure robust error analysis, each experiment was conducted in triplicate. The results are the mean ± S.D., with error bars representing the degree of variability within the data set. All statistical analyses were performed utilizing SPSS software (version 24.0; SPSS, Chicago, IL), ensuring rigorous and standardized data interpretation.

## Data Availability

All data generated during this study are included in the article and its supplemental material.

## AFTER EPUB

*[This article was published on 10 July 2025 with the incorrect Fig. 7. The figure was updated in the current version, posted on 12 August 2025.]*

## SUPPLEMENTAL MATERIAL

Supplemental material aem.00601-25-s0001.docx Table S1; Fig. S1.

ASM does not own the copyrights to Supplemental Material that may be linked to, or accessed through, an article. The authors have granted ASM a non-exclusive, world-wide license to publish the Supplemental Material files. Please contact the corresponding author directly for reuse.
