# Supplementary material for: Optimized genetic circuitry and reporters for sensitive whole-cell arsenic biosensors: advancing environmental monitoring
Source: Appl Environ Microbiol. 2025 Jul 10;91(8):e00601-25. doi: 10.1128/aem.00601-25 (PMC12366313; doi:10.1128/aem.00601-25)
Supplement: Supplemental material — Table S1; Fig. S1. [file aem.00601-25-s0001.docx]

**TABLE S1** DNA sequence of arsenic biosensing elements used in this study.

| Vectors | | Arsenic sensory element | sites |
| --- | --- | --- | --- |
| pnK12-R | AGATCTCTGCACTTACACATTCGTTAAGTCATATATGTTTTTGACTTATCCGCTTCGAAGAGAGACACTACCTGCAACAATCAGGAGCGCAATATGTCATTTCTGTTACCCATCCAATTGTTCAAAATTCTTGCTGATGAAACCCGTCTGGGCATCGTTTTACTGCTCAGCGAACTGGGAGAGTTATGCGTCTGCGATCTCTGCACTGCTCTCGACCAGTCGCAGCCCAAGATCTCCCGCCACCTGGCATTGCTGCGTGAAAGCGGGCTATTGCTGGACCGCAAGCAAGGTAAGTGGGTTCATTACCGCTTATCACCGCATATTCCAGCATGGGCGGCGAAAATTATTGATGAGGCCTGGCGATGTGAACAGGAAAAGGTTCAGGCGATTGTCCGCAACCTGGCTCGACAAAACTGTTCCGGGGACAGTAAGAACATTTGCAGTTAATCTAGAAATAATTTTGTTTAACTTTAAGAAGGAGATATACATATGGTCTCTAAAGGCGAGGAAGACAACATGGCAATCATCAAAGAGTTCATGCGTTTCAAAGTGCACATGGAGGGTAGCGTCAACGGTCACGAATTTGAAATCGAAGGTGAGGGTGAAGGTCGCCCGTACGAAGGTACCCAAACCGCTAAACTGAAAGTGACGAAAGGTGGTCCGCTGCCATTCGCATGGGATATCCTGTCTCCACAGTTCATGTACGGTTCTAAAGCGTACGTGAAACACCCGGCTGACATTCCTGACTACCTGAAACTGTCCTTCCCGGAAGGTTTCAAATGGGAACGTGTGATGAACTTCGAGGACGGTGGCGTAGTTACTGTTACCCAGGACTCTTCCCTGCAGGATGGTGAGTTTATCTACAAGGTTAAACTGCGTGGCACTAACTTTCCGTCCGACGGCCCGGTTATGCAGAAGAAGACTATGGGCTGGGAAGCATCTAGCGAACGTATGTATCCGGAAGATGGTGCTCTGAAAGGCGAAATCAAACAGCGTCTGAAACTGAAAGACGGCGGCCATTATGATGCGGAAGTTAAGACGACCTACAAAGCCAAGAAACCGGTTCAGCTGCCGGGCGCCTATAATGTAAACATCAAACTGGATATTACCTCCCACAACGAAGATTACACCATTGTAGAACAATATGAACGCGCGGAAGGCCGCCATAGCACCGGCGGCATGGACGAACTGTACAAAtaaGAGCTC | | *Bgl* II  *Sac* I |
| pnK12-ABS-R | AGATCTCTGCACTTACACATTCGTTAAGTCATATATGTTTTTGACTTATCCGCTTCGAAGAGAGACACTACCTGCAACAATCAGGAGCGCAATATGTCATTTCTGTTACCCATCCAATTGTTCAAAATTCTTGCTGATGAAACCCGTCTGGGCATCGTTTTACTGCTCAGCGAACTGGGAGAGTTATGCGTCTGCGATCTCTGCACTGCTCTCGACCAGTCGCAGCCCAAGATCTCCCGCCACCTGGCATTGCTGCGTGAAAGCGGGCTATTGCTGGACCGCAAGCAAGGTAAGTGGGTTCATTACCGCTTATCACCGCATATTCCAGCATGGGCGGCGAAAATTATTGATGAGGCCTGGCGATGTGAACAGGAAAAGGTTCAGGCGATTGTCCGCAACCTGGCTCGACAAAACTGTTCCGGGGACAGTAAGAACATTTGCAGTTAATACACATTCGTTAAGTCATATATGTTTTTGACTTATCTAGAAATAATTTTGTTTAACTTTAAGAAGGAGATATACATATGGTCTCTAAAGGCGAGGAAGACAACATGGCAATCATCAAAGAGTTCATGCGTTTCAAAGTGCACATGGAGGGTAGCGTCAACGGTCACGAATTTGAAATCGAAGGTGAGGGTGAAGGTCGCCCGTACGAAGGTACCCAAACCGCTAAACTGAAAGTGACGAAAGGTGGTCCGCTGCCATTCGCATGGGATATCCTGTCTCCACAGTTCATGTACGGTTCTAAAGCGTACGTGAAACACCCGGCTGACATTCCTGACTACCTGAAACTGTCCTTCCCGGAAGGTTTCAAATGGGAACGTGTGATGAACTTCGAGGACGGTGGCGTAGTTACTGTTACCCAGGACTCTTCCCTGCAGGATGGTGAGTTTATCTACAAGGTTAAACTGCGTGGCACTAACTTTCCGTCCGACGGCCCGGTTATGCAGAAGAAGACTATGGGCTGGGAAGCATCTAGCGAACGTATGTATCCGGAAGATGGTGCTCTGAAAGGCGAAATCAAACAGCGTCTGAAACTGAAAGACGGCGGCCATTATGATGCGGAAGTTAAGACGACCTACAAAGCCAAGAAACCGGTTCAGCTGCCGGGCGCCTATAATGTAAACATCAAACTGGATATTACCTCCCACAACGAAGATTACACCATTGTAGAACAATATGAACGCGCGGAAGGCCGCCATAGCACCGGCGGCATGGACGAACTGTACAAAtaaGAGCTC | | *Bgl* II  *Sac* I |
| pnK12-ABS-R-GlpF | AGATCTCTGCACTTACACATTCGTTAAGTCATATATGTTTTTGACTTATCCGCTTCGAAGAGAGACACTACCTGCAACAATCAGGAGCGCAATATGTCATTTCTGTTACCCATCCAATTGTTCAAAATTCTTGCTGATGAAACCCGTCTGGGCATCGTTTTACTGCTCAGCGAACTGGGAGAGTTATGCGTCTGCGATCTCTGCACTGCTCTCGACCAGTCGCAGCCCAAGATCTCCCGCCACCTGGCATTGCTGCGTGAAAGCGGGCTATTGCTGGACCGCAAGCAAGGTAAGTGGGTTCATTACCGCTTATCACCGCATATTCCAGCATGGGCGGCGAAAATTATTGATGAGGCCTGGCGATGTGAACAGGAAAAGGTTCAGGCGATTGTCCGCAACCTGGCTCGACAAAACTGTTCCGGGGACAGTAAGAACATTTGCAGTTAATACACATTCGTTAAGTCATATATGTTTTTGACTTATCTAGAAATAATTTTGTTTAACTTTAAGAAGGAGATATACATATGGTCTCTAAAGGCGAGGAAGACAACATGGCAATCATCAAAGAGTTCATGCGTTTCAAAGTGCACATGGAGGGTAGCGTCAACGGTCACGAATTTGAAATCGAAGGTGAGGGTGAAGGTCGCCCGTACGAAGGTACCCAAACCGCTAAACTGAAAGTGACGAAAGGTGGTCCGCTGCCATTCGCATGGGATATCCTGTCTCCACAGTTCATGTACGGTTCTAAAGCGTACGTGAAACACCCGGCTGACATTCCTGACTACCTGAAACTGTCCTTCCCGGAAGGTTTCAAATGGGAACGTGTGATGAACTTCGAGGACGGTGGCGTAGTTACTGTTACCCAGGACTCTTCCCTGCAGGATGGTGAGTTTATCTACAAGGTTAAACTGCGTGGCACTAACTTTCCGTCCGACGGCCCGGTTATGCAGAAGAAGACTATGGGCTGGGAAGCATCTAGCGAACGTATGTATCCGGAAGATGGTGCTCTGAAAGGCGAAATCAAACAGCGTCTGAAACTGAAAGACGGCGGCCATTATGATGCGGAAGTTAAGACGACCTACAAAGCCAAGAAACCGGTTCAGCTGCCGGGCGCCTATAATGTAAACATCAAACTGGATATTACCTCCCACAACGAAGATTACACCATTGTAGAACAATATGAACGCGCGGAAGGCCGCCATAGCACCGGCGGCATGGACGAACTGTACAAAtaaGAGCTCTTATTGTGGTGGCGGTCGATATTCGCACTGGCAAAAAAACGTGCTTGAATATCTGTTGAAACCCTTTAACAAAGCACAGGTACCAGGAGGCGTTGCGCGAACGATGAGCCAGACTTCCACTCTGAAAGGTCAATGTATTGCTGAATTTCTGGGCACCGGTCTGCTTATTTTCTTCGGCGTTGGCTGTGTTGCGGCGCTGAAAGTTGCGGGCGCGTCTTTCGGACAGTGGGAAATCAGCGTTATCTGGGGTCTGGGCGTTGCGATGGCGATCTACCTGACCGCGGGCGTTTCTGGTGCGCACCTGAACCCGGCGGTTACCATCGCGCTGTGGCTGTTCGCGTGCTTCGATAAACGTAAAGTTATCCCGTTCATCGTTTCTCAGGTTGCGGGTGCTTTCTGCGCGGCGGCGCTGGTTTACGGCCTGTACTACAACCTGTTCTTCGATTTCGAACAGACCCACCACATCGTTCGTGGTAGCGTTGAATCCGTTGATCTGGCGGGCACCTTCAGCACCTACCCGAACCCGCACATCAACTTCGTTCAGGCGTTCGCGGTTGAAATGGTTATCACCGCGATCCTGATGGGCCTGATCCTGGCGCTGACCGATGACGGCAACGGTGTTCCGCGTGGCCCGCTGGCGCCGCTGCTGATCGGCCTGCTGATCGCGGTTATCGGTGCATCTATGGGTCCGCTGACCGGTTTCGCGATGAACCCGGCGCGTGATTTCGGCCCGAAAGTTTTCGCGTGGCTGGCGGGTTGGGGTAACGTTGCGTTCACCGGCGGTCGTGATATTCCGTATTTCTTAGTTCCGCTGTTCGGCCCGATTGTTGGCGCTATCGTTGGTGCATTCGCTTATCGTAAACTGATCGGCCGTCACCTGCCGTGCGATATCTGCGTTGTTGAAGAAAAAGAAACCACCACCCCGTCTGAACAGAAAGCGAGCCTGtaaAAGCTT | | *Bgl* II  *Hin*d III |
| pK12-R | AGATCTTTAACTGCAAATGTTCTTACTGTCCCCGGAACAGTTTTGTCGAGCCAGGTTGCGGACAATCGCCTGAACCTTTTCCTGTTCACATCGCCAGGCCTCATCAATAATTTTCGCCGCCCATGCTGGAATATGCGGTGATAAGCGGTAATGAACCCACTTACCTTGCTTGCGGTCCAGCAATAGCCCGCTTTCACGCAGCAATGCCAGGTGGCGGGAGATCTTGGGCTGCGACTGGTCGAGAGCAGTGCAGAGATCGCAGACGCATAACTCTCCCAGTTCGCTGAGCAGTAAAACGATGCCCAGACGGGTTTCATCAGCAAGAATTTTGAACAATTGGATGGGTAACAGAAATGACATCGTTCGCGCAACGCCTCCTGGTACCTGTGCTTTGTTAAAGGGTTTCAACAGATATTCAACTGCACTTACACATTCGTTAAGTCATATATGTTTTTGACTTATCCGCTTCGAAGAGAGACACTACCTGCAACAATCAGGAGCGCAATCATATGGTCTCTAAAGGCGAGGAAGACAACATGGCAATCATCAAAGAGTTCATGCGTTTCAAAGTGCACATGGAGGGTAGCGTCAACGGTCACGAATTTGAAATCGAAGGTGAGGGTGAAGGTCGCCCGTACGAAGGTACCCAAACCGCTAAACTGAAAGTGACGAAAGGTGGTCCGCTGCCATTCGCATGGGATATCCTGTCTCCACAGTTCATGTACGGTTCTAAAGCGTACGTGAAACACCCGGCTGACATTCCTGACTACCTGAAACTGTCCTTCCCGGAAGGTTTCAAATGGGAACGTGTGATGAACTTCGAGGACGGTGGCGTAGTTACTGTTACCCAGGACTCTTCCCTGCAGGATGGTGAGTTTATCTACAAGGTTAAACTGCGTGGCACTAACTTTCCGTCCGACGGCCCGGTTATGCAGAAGAAGACTATGGGCTGGGAAGCATCTAGCGAACGTATGTATCCGGAAGATGGTGCTCTGAAAGGCGAAATCAAACAGCGTCTGAAACTGAAAGACGGCGGCCATTATGATGCGGAAGTTAAGACGACCTACAAAGCCAAGAAACCGGTTCAGCTGCCGGGCGCCTATAATGTAAACATCAAACTGGATATTACCTCCCACAACGAAGATTACACCATTGTAGAACAATATGAACGCGCGGAAGGCCGCCATAGCACCGGCGGCATGGACGAACTGTACAAAtaaGAGCTC | | *Bgl* II  *Sac* I |
| pJ23119-R | AGATCTTTAACTGCAAATGTTCTTACTGTCCCCGGAACAGTTTTGTCGAGCCAGGTTGCGGACAATCGCCTGAACCTTTTCCTGTTCACATCGCCAGGCCTCATCAATAATTTTCGCCGCCCATGCTGGAATATGCGGTGATAAGCGGTAATGAACCCACTTACCTTGCTTGCGGTCCAGCAATAGCCCGCTTTCACGCAGCAATGCCAGGTGGCGGGAGATCTTGGGCTGCGACTGGTCGAGAGCAGTGCAGAGATCGCAGACGCATAACTCTCCCAGTTCGCTGAGCAGTAAAACGATGCCCAGACGGGTTTCATCAGCAAGAATTTTGAACAATTGGATGGGTAACAGAAATGACATCCTAGCATTATACCTAGGACTGAGCTAGCTGTCAACTGCACTTACACATTCGTTAAGTCATATATGTTTTTGACTTATCCGCTTCGAAGAGAGACACTACCTGCAACAATCAGGAGCGCAATCATATGGTCTCTAAAGGCGAGGAAGACAACATGGCAATCATCAAAGAGTTCATGCGTTTCAAAGTGCACATGGAGGGTAGCGTCAACGGTCACGAATTTGAAATCGAAGGTGAGGGTGAAGGTCGCCCGTACGAAGGTACCCAAACCGCTAAACTGAAAGTGACGAAAGGTGGTCCGCTGCCATTCGCATGGGATATCCTGTCTCCACAGTTCATGTACGGTTCTAAAGCGTACGTGAAACACCCGGCTGACATTCCTGACTACCTGAAACTGTCCTTCCCGGAAGGTTTCAAATGGGAACGTGTGATGAACTTCGAGGACGGTGGCGTAGTTACTGTTACCCAGGACTCTTCCCTGCAGGATGGTGAGTTTATCTACAAGGTTAAACTGCGTGGCACTAACTTTCCGTCCGACGGCCCGGTTATGCAGAAGAAGACTATGGGCTGGGAAGCATCTAGCGAACGTATGTATCCGGAAGATGGTGCTCTGAAAGGCGAAATCAAACAGCGTCTGAAACTGAAAGACGGCGGCCATTATGATGCGGAAGTTAAGACGACCTACAAAGCCAAGAAACCGGTTCAGCTGCCGGGCGCCTATAATGTAAACATCAAACTGGATATTACCTCCCACAACGAAGATTACACCATTGTAGAACAATATGAACGCGCGGAAGGCCGCCATAGCACCGGCGGCATGGACGAACTGTACAAAtaaGAGCTC | | *Bgl* II  *Sac* I |
| pJ23119-R-GlpF | AGATCTTTAACTGCAAATGTTCTTACTGTCCCCGGAACAGTTTTGTCGAGCCAGGTTGCGGACAATCGCCTGAACCTTTTCCTGTTCACATCGCCAGGCCTCATCAATAATTTTCGCCGCCCATGCTGGAATATGCGGTGATAAGCGGTAATGAACCCACTTACCTTGCTTGCGGTCCAGCAATAGCCCGCTTTCACGCAGCAATGCCAGGTGGCGGGAGATCTTGGGCTGCGACTGGTCGAGAGCAGTGCAGAGATCGCAGACGCATAACTCTCCCAGTTCGCTGAGCAGTAAAACGATGCCCAGACGGGTTTCATCAGCAAGAATTTTGAACAATTGGATGGGTAACAGAAATGACATCCTAGCATTATACCTAGGACTGAGCTAGCTGTCAACTGCACTTACACATTCGTTAAGTCATATATGTTTTTGACTTATCCGCTTCGAAGAGAGACACTACCTGCAACAATCAGGAGCGCAATCATATGGTCTCTAAAGGCGAGGAAGACAACATGGCAATCATCAAAGAGTTCATGCGTTTCAAAGTGCACATGGAGGGTAGCGTCAACGGTCACGAATTTGAAATCGAAGGTGAGGGTGAAGGTCGCCCGTACGAAGGTACCCAAACCGCTAAACTGAAAGTGACGAAAGGTGGTCCGCTGCCATTCGCATGGGATATCCTGTCTCCACAGTTCATGTACGGTTCTAAAGCGTACGTGAAACACCCGGCTGACATTCCTGACTACCTGAAACTGTCCTTCCCGGAAGGTTTCAAATGGGAACGTGTGATGAACTTCGAGGACGGTGGCGTAGTTACTGTTACCCAGGACTCTTCCCTGCAGGATGGTGAGTTTATCTACAAGGTTAAACTGCGTGGCACTAACTTTCCGTCCGACGGCCCGGTTATGCAGAAGAAGACTATGGGCTGGGAAGCATCTAGCGAACGTATGTATCCGGAAGATGGTGCTCTGAAAGGCGAAATCAAACAGCGTCTGAAACTGAAAGACGGCGGCCATTATGATGCGGAAGTTAAGACGACCTACAAAGCCAAGAAACCGGTTCAGCTGCCGGGCGCCTATAATGTAAACATCAAACTGGATATTACCTCCCACAACGAAGATTACACCATTGTAGAACAATATGAACGCGCGGAAGGCCGCCATAGCACCGGCGGCATGGACGAACTGTACAAAtaaGAGCTCTTATTGTGGTGGCGGTCGATATTCGCACTGGCAAAAAAACGTGCTTGAATATCTGTTGAAACCCTTTAACAAAGCACAGGTACCAGGAGGCGTTGCGCGAACGATGAGCCAGACTTCCACTCTGAAAGGTCAATGTATTGCTGAATTTCTGGGCACCGGTCTGCTTATTTTCTTCGGCGTTGGCTGTGTTGCGGCGCTGAAAGTTGCGGGCGCGTCTTTCGGACAGTGGGAAATCAGCGTTATCTGGGGTCTGGGCGTTGCGATGGCGATCTACCTGACCGCGGGCGTTTCTGGTGCGCACCTGAACCCGGCGGTTACCATCGCGCTGTGGCTGTTCGCGTGCTTCGATAAACGTAAAGTTATCCCGTTCATCGTTTCTCAGGTTGCGGGTGCTTTCTGCGCGGCGGCGCTGGTTTACGGCCTGTACTACAACCTGTTCTTCGATTTCGAACAGACCCACCACATCGTTCGTGGTAGCGTTGAATCCGTTGATCTGGCGGGCACCTTCAGCACCTACCCGAACCCGCACATCAACTTCGTTCAGGCGTTCGCGGTTGAAATGGTTATCACCGCGATCCTGATGGGCCTGATCCTGGCGCTGACCGATGACGGCAACGGTGTTCCGCGTGGCCCGCTGGCGCCGCTGCTGATCGGCCTGCTGATCGCGGTTATCGGTGCATCTATGGGTCCGCTGACCGGTTTCGCGATGAACCCGGCGCGTGATTTCGGCCCGAAAGTTTTCGCGTGGCTGGCGGGTTGGGGTAACGTTGCGTTCACCGGCGGTCGTGATATTCCGTATTTCTTAGTTCCGCTGTTCGGCCCGATTGTTGGCGCTATCGTTGGTGCATTCGCTTATCGTAAACTGATCGGCCGTCACCTGCCGTGCGATATCTGCGTTGTTGAAGAAAAAGAAACCACCACCCCGTCTGAACAGAAAGCGAGCCTGtaaAAGCTT | | *Bgl* II  *Hin*d III |
| pK12-ind | AGATCTTTAACTGCAAATGTTCTTACTGTCCCCGGAACAGTTTTGTCGAGCCAGGTTGCGGACAATCGCCTGAACCTTTTCCTGTTCACATCGCCAGGCCTCATCAATAATTTTCGCCGCCCATGCTGGAATATGCGGTGATAAGCGGTAATGAACCCACTTACCTTGCTTGCGGTCCAGCAATAGCCCGCTTTCACGCAGCAATGCCAGGTGGCGGGAGATCTTGGGCTGCGACTGGTCGAGAGCAGTGCAGAGATCGCAGACGCATAACTCTCCCAGTTCGCTGAGCAGTAAAACGATGCCCAGACGGGTTTCATCAGCAAGAATTTTGAACAATTGGATGGGTAACAGAAATGACATCGTTCGCGCAACGCCTCCTGGTACCTGTGCTTTGTTAAAGGGTTTCAACAGATATTCAACTGCACTTACACATTCGTTAAGTCATATATGTTTTTGACTTATCCGCTTCGAAGAGAGACACTACCTGCAACAATCAGGAGCGCAATCATATGACCCTGCAGGAAACCTCTGTTCTGGAACCGACCCTGCAGGGCACCACCACGCTGCCGGGTCTGCTGGCTCAGCGCGTAGCTGAACACCCGGAAGCTATCGCAGTTGCTTACCGTGATGACAAACTGACCTTCCGTGAACTGGCTTCTCGCTCGGCCGCGCTGGCGGACTACCTGGAACACCTGGGTGTCTCCGCGGACGACTGTGTTGGTCTGTTCGTAGAACCGTCTATCGACCTGATGGTTGGCGCGTGGGGCATCCTGAACGCCGGCGCGGCATACCTGCCGCTGAGCCCGGAATACCCGGAAGATCGCCTGCGTTACATGATCGAAAACAGCGAAACTAAAATTATCCTGGCCCAGCAGCGCCTGGTTAGCCGCCTGCGTGAACTCGCCCCGAAAGACGTTACCATTGTTACTTTACGTGAATCTGAAGCCTTTGTGCGTCCGGAAGGCACGGAAGCGCCGGCGGCACGCTCCGCTCGTCCGGACACCCTGGCCTACGTGATCTATACTTCCGGTAGCACCGGAAAACCGAAAGGTGTTATGATTGAACACCGCTCTATCGTTAACCAACTGGGCTGGCTGCGTGAAACCTACGCCATCGACCGTTCTAAAGTAATTCTGCAGAAAACTCCGATGTCGTTCGACGCTGCTCAGTGGGAAATTCTGTCTCCGGCTAACGGCGCGACCGTTGTTATGGGCGCACCGGGTGTCTACGCGGACCCGGAAGGTCTGATCGAAACCATCGTGAAACATAACGTTACCACTCTGCAGTGCGTTCCGACCCTGCTGCAGGGTCTGATTGATACCGAGAAATTCCCGGAATGCGTAAGTCTGCAGCAGATTTTCTCCGGCGGTGAAGCGCTGTCCCGCCTGCTGGCCATCCAGACCACTCAAGAAATGCCGGGCCGTGCCCTGATCAACGTTTACGGCCCTACAGAAACCACGATTAACAGCTCCTCTTTTCCGGTAGATCCGGCAGACCTCGATGAAGGCCCGCAGAGCATCAGCATCGGTAGCCCGGTACACGGTACCACCTACCACATCCTGGACAAAGAAACCCTCAAACCGGTCGGCGTCGGCGAAATTGGTGAACTGTATATCGGCGGCATTCAGCTGGCTCGCGGCTACCTGCACCGTGACGACCTGACTGCCGAACGCTTCCTGGAGATTGAACTGGAAGAAGGTGCGGAACCGGTTCGCTTGTATAAAACTGGTGATCTGGGTCAGTGGAACAACGATGGCACGGTTCAGTTCGCTGGTCGTGCAGACAACCAAGTGAAACTGCGTGGTTATCGCGTTGAACTTGATGAAATTAGCCTGGCGATTGAAAATCACGATTGGGTTCGTAACGCTGCAGTGATCGTGAAAAATGACGGTCGTACTGGTTTTCAGAACCTGATCGCTTGTATTGAACTGAGTGAAAAAGAAGCAGCACTGATGGATCAGGGTAATCATGGTTCACATCATGCTTCTAAAAAATCCAAACTGCAGGTGAAGGCCCAGCTGAGCAATCCGGGTCTGCGTGATGATGCGGAATTAGCCGCTCGCCCGGCCTTCGACCTGGAAGGTGCAGAGCCAACCCCTGAACAACGTGCGCGTGTTTTTGCGCGCAAAACGTACCGTTTCTACGAGGGCGGCGCGGTGACGCAGGCTGACTTGCTGGGCCTGCTGGGTGCGACCGTGACCGCGGGTTATTCTCGTAAAGCTGCCGATCTGGCACCGGCGGAACTGGGCCAGATTCTGCGTTGGTTCGGCCAGTACATTTCTGAAGAACGTCTGTTACCAAAATATGGTTACGCTTCTCCTGGTGCTCTGTACGCAACCCAAATGTACTTCGAACTGGAAGGTGTTGGCGGTTTGAAACCGGGTTATTATTACTACCAACCGGTTCGTCACCAGCTCGTGTTGATTTCTGAACGTGAAGCCACCGGTAAAGCCACCGCACAGATCCACTTCATTGGCAAAAAGAGTGGCATTGAACCGGTTTATAAAAATAACATCTTGGAAGTTCTTGAAATTGAAACTGGTCACATGGTTGGTCTGTTCGAACAGATCCTGCCGGCATACGGCCTCGATATTCATGATCGTGCGTACGAACCGGCTGTTAAGGATCTGCTGGACGTTGCGGATGAGGACTACTACCTGGGCACTTTCGAACTGGTTCCGCACGCGGGTGCACGCGACGATCAGGCGGAAGTCTATGTACAGACCCACGGTGGCAAAGTAGCTGGTCTGCCGGAAGGTCAGTATCGCTATGAAAATGGTGAGTTGACTCGCTTCAGCGACGACATTGTTCTGAAAAAACACGTTATTGCTATCAATCAGTCCGTCTACCAAGCTGCGTCCTTTGGTATCAGCGTTTACTCGCGTGCAGAAGAAGAATGGTTAAAGTACATCACGCTGGGTAAAAAACTGCAACATCTGATGATGAACGGTCTTAACCTGGGCTTTATGTCCAGCGGTTACTCCTCTAAAACCGGTAATCCGCTGCCGGCGTCCCGCCGTATGGATGCTGTTCTGGGTGCTAACGGCGTGGACAGCGCGCCAATGTATTTCTTTGTTGGTGGCCGCATTTCGGACGAACAGATCGGTCACGAAGGTATGCGTGAAGATTCCGTGCACATGCGCGGTCCGGCTGAACTGATCCGTGACGACCTGGTTAGCTTCCTGCCGGACTATATGATCCCGAACCGTGTCGTGGTATTCGATCGTCTGCCACTGAGCGCGAACGGTAAAATCGACGTTAAAGCACTGGCGGCCTCTGATCAGGTGAACGCGGAGCTGGTTGAACGTCCGTTTGTCGCACCGCGTACTGAAACAGAAAAAGAAATTGCTGCTGTGTGGGAAAAAGCACTGCGTCGTGAAAACGCCTCTGTGCAAGATGACTTCTTTGAGAGCGGCGGGAACTCCCTGATTGCGGTTGGTCTGGTTCGTGAACTGAACGCGCGTTTAGGCGTCAGCTTACCGCTGCAGTCTGTACTGGAGTCCCCGACCATTGAAAAACTGGCACGTCGCCTGGAACGTGAAGTGGCGCAGGAATCAAGCCGTTTTGTTCGTTTACACGCAGAAACTGGTAAAGCGCGTCCGGTGATTTGCTGGCCGGGCCTGGGCGGTTATCCTATGAACCTGCGTAGCCTGGCAGGTGAAATCGGCCTGGGCCGTAGCTTCTATGGTGTGCAGAGCTACGGCATTAACGAAGGTGAAACCCCGTATGAAACTATCACCGAGATGGCGAAAAAAGATATCGAGGCTCTGAAAGAAATCCAGCCGGCGGGGCCGTATACCCTGTGGGGCTACTCTTTCGGTGCGCGCGTTGCGTTTGAAACCGCCTATCAGCTGGAACAGGCTGGTGAAAAAGTGGATAACCTGTTCCTGATTGCACCGGGCTCACCGAAAGTTCGCGCTGAGAACGGTAAAGTTTGGGGTCGCGAAGCGAGCTTCGCAAACCGCGGATACACGACCATCCTGTTTTCTGTGTTTACTGGCACCATTTCCGGCCCAGACCTGGACCGTTGCCTGGAAACCGTGACTGATGAAGCATCCTTCGCTGAGTTCATCTCAGAGCTGAAAGGTATTGACGTTGACCTGGCGCGTCGCATCATCAGCGTAGTGGGCCAGACGTACGAGTTCGAATACTCTTTCCATGAACTGGCGGAGCGTACCCTGCAGGCGCCGATCTCTATCTTCAAAGCCGTGGGCGATGACTACTCCTTCCTGGAAAATAGCTCTGGCTATTCCGCGGAACCGCCGACCGTGATCGATCTGGATGCGGATCATTACAGCCTGCTGCGTGAAGATATCGGTGAACTGGTAAAACATATCCGCTATCTCCTGGGTGAATAATTAAGGAGGTAAAAAAAATGCGTGCGATGAACGATCGTCTGCCGTCCTTCTGCACCCCGCTGGATGATCGTTGGCCGCTGCCGGTTGCGCTGCCGGGCGTGCAGCTGCGTTCGACCCGCTTCGATCCGGCGCTGCTGCAGCCGGGCGATTTCGCGCTGGCGGGCATCCAGCCGCCGGCGAACATCCTGCGTGCGGTGGCGAAACGTCAGGCGGAGTTCCTGGCGGGTCGTCTGTGCGCGCGTGCGGCGCTGTTCGCGCTGGATGGTCGTGCGCAGACTCCGGCTGTTGGCGAAGATCGTGCGCCGGTTTGGCCGGCGGCGATCTCCGGCTCTATCACCCACGGCGATCGCTGGGCGGCGGCGCTGGTTGCGGCGCGCGGCGATTGGCGTGGTCTGGGTCTGGATGTTGAAACCCTGCTGGAAGCGGAACGTGCGCGTTACCTGCACGGCGAAATCCTGACCGAAGGCGAACGTCTGCGTTTCGCGGATGATCTGGAACGTCGTACCGGCCTGCTGGTGACCCTGGCGTTCTCCCTGAAAGAAAGCCTGTTCAAAGCGCTGTACCCGCTGGTTGGCAAACGTTTCTACTTCGAACACGCAGAACTGCTGGAATGGCGTGCGGACGGTCAAGCGCGTCTGCGTCTGCTGACCGATCTGAGCCCGGAATGGCGTCACGGTTCTGAACTGGATGCGCAGTTCGCGGTTCTGGACGGCCGTCTGCTGTCTCTGGTTGCGGTTGGTGCGtaaGAGCTC | | *Bgl* II  *Sac* I |
| pnK12-ind | AGATCTCTGCACTTACACATTCGTTAAGTCATATATGTTTTTGACTTATCCGCTTCGAAGAGAGACACTACCTGCAACAATCAGGAGCGCAATATGTCATTTCTGTTACCCATCCAATTGTTCAAAATTCTTGCTGATGAAACCCGTCTGGGCATCGTTTTACTGCTCAGCGAACTGGGAGAGTTATGCGTCTGCGATCTCTGCACTGCTCTCGACCAGTCGCAGCCCAAGATCTCCCGCCACCTGGCATTGCTGCGTGAAAGCGGGCTATTGCTGGACCGCAAGCAAGGTAAGTGGGTTCATTACCGCTTATCACCGCATATTCCAGCATGGGCGGCGAAAATTATTGATGAGGCCTGGCGATGTGAACAGGAAAAGGTTCAGGCGATTGTCCGCAACCTGGCTCGACAAAACTGTTCCGGGGACAGTAAGAACATTTGCAGTTAATCTAGAAATAATTTTGTTTAACTTTAAGAAGGAGATATACATATGACCCTGCAGGAAACCTCTGTTCTGGAACCGACCCTGCAGGGCACCACCACGCTGCCGGGTCTGCTGGCTCAGCGCGTAGCTGAACACCCGGAAGCTATCGCAGTTGCTTACCGTGATGACAAACTGACCTTCCGTGAACTGGCTTCTCGCTCGGCCGCGCTGGCGGACTACCTGGAACACCTGGGTGTCTCCGCGGACGACTGTGTTGGTCTGTTCGTAGAACCGTCTATCGACCTGATGGTTGGCGCGTGGGGCATCCTGAACGCCGGCGCGGCATACCTGCCGCTGAGCCCGGAATACCCGGAAGATCGCCTGCGTTACATGATCGAAAACAGCGAAACTAAAATTATCCTGGCCCAGCAGCGCCTGGTTAGCCGCCTGCGTGAACTCGCCCCGAAAGACGTTACCATTGTTACTTTACGTGAATCTGAAGCCTTTGTGCGTCCGGAAGGCACGGAAGCGCCGGCGGCACGCTCCGCTCGTCCGGACACCCTGGCCTACGTGATCTATACTTCCGGTAGCACCGGAAAACCGAAAGGTGTTATGATTGAACACCGCTCTATCGTTAACCAACTGGGCTGGCTGCGTGAAACCTACGCCATCGACCGTTCTAAAGTAATTCTGCAGAAAACTCCGATGTCGTTCGACGCTGCTCAGTGGGAAATTCTGTCTCCGGCTAACGGCGCGACCGTTGTTATGGGCGCACCGGGTGTCTACGCGGACCCGGAAGGTCTGATCGAAACCATCGTGAAACATAACGTTACCACTCTGCAGTGCGTTCCGACCCTGCTGCAGGGTCTGATTGATACCGAGAAATTCCCGGAATGCGTAAGTCTGCAGCAGATTTTCTCCGGCGGTGAAGCGCTGTCCCGCCTGCTGGCCATCCAGACCACTCAAGAAATGCCGGGCCGTGCCCTGATCAACGTTTACGGCCCTACAGAAACCACGATTAACAGCTCCTCTTTTCCGGTAGATCCGGCAGACCTCGATGAAGGCCCGCAGAGCATCAGCATCGGTAGCCCGGTACACGGTACCACCTACCACATCCTGGACAAAGAAACCCTCAAACCGGTCGGCGTCGGCGAAATTGGTGAACTGTATATCGGCGGCATTCAGCTGGCTCGCGGCTACCTGCACCGTGACGACCTGACTGCCGAACGCTTCCTGGAGATTGAACTGGAAGAAGGTGCGGAACCGGTTCGCTTGTATAAAACTGGTGATCTGGGTCAGTGGAACAACGATGGCACGGTTCAGTTCGCTGGTCGTGCAGACAACCAAGTGAAACTGCGTGGTTATCGCGTTGAACTTGATGAAATTAGCCTGGCGATTGAAAATCACGATTGGGTTCGTAACGCTGCAGTGATCGTGAAAAATGACGGTCGTACTGGTTTTCAGAACCTGATCGCTTGTATTGAACTGAGTGAAAAAGAAGCAGCACTGATGGATCAGGGTAATCATGGTTCACATCATGCTTCTAAAAAATCCAAACTGCAGGTGAAGGCCCAGCTGAGCAATCCGGGTCTGCGTGATGATGCGGAATTAGCCGCTCGCCCGGCCTTCGACCTGGAAGGTGCAGAGCCAACCCCTGAACAACGTGCGCGTGTTTTTGCGCGCAAAACGTACCGTTTCTACGAGGGCGGCGCGGTGACGCAGGCTGACTTGCTGGGCCTGCTGGGTGCGACCGTGACCGCGGGTTATTCTCGTAAAGCTGCCGATCTGGCACCGGCGGAACTGGGCCAGATTCTGCGTTGGTTCGGCCAGTACATTTCTGAAGAACGTCTGTTACCAAAATATGGTTACGCTTCTCCTGGTGCTCTGTACGCAACCCAAATGTACTTCGAACTGGAAGGTGTTGGCGGTTTGAAACCGGGTTATTATTACTACCAACCGGTTCGTCACCAGCTCGTGTTGATTTCTGAACGTGAAGCCACCGGTAAAGCCACCGCACAGATCCACTTCATTGGCAAAAAGAGTGGCATTGAACCGGTTTATAAAAATAACATCTTGGAAGTTCTTGAAATTGAAACTGGTCACATGGTTGGTCTGTTCGAACAGATCCTGCCGGCATACGGCCTCGATATTCATGATCGTGCGTACGAACCGGCTGTTAAGGATCTGCTGGACGTTGCGGATGAGGACTACTACCTGGGCACTTTCGAACTGGTTCCGCACGCGGGTGCACGCGACGATCAGGCGGAAGTCTATGTACAGACCCACGGTGGCAAAGTAGCTGGTCTGCCGGAAGGTCAGTATCGCTATGAAAATGGTGAGTTGACTCGCTTCAGCGACGACATTGTTCTGAAAAAACACGTTATTGCTATCAATCAGTCCGTCTACCAAGCTGCGTCCTTTGGTATCAGCGTTTACTCGCGTGCAGAAGAAGAATGGTTAAAGTACATCACGCTGGGTAAAAAACTGCAACATCTGATGATGAACGGTCTTAACCTGGGCTTTATGTCCAGCGGTTACTCCTCTAAAACCGGTAATCCGCTGCCGGCGTCCCGCCGTATGGATGCTGTTCTGGGTGCTAACGGCGTGGACAGCGCGCCAATGTATTTCTTTGTTGGTGGCCGCATTTCGGACGAACAGATCGGTCACGAAGGTATGCGTGAAGATTCCGTGCACATGCGCGGTCCGGCTGAACTGATCCGTGACGACCTGGTTAGCTTCCTGCCGGACTATATGATCCCGAACCGTGTCGTGGTATTCGATCGTCTGCCACTGAGCGCGAACGGTAAAATCGACGTTAAAGCACTGGCGGCCTCTGATCAGGTGAACGCGGAGCTGGTTGAACGTCCGTTTGTCGCACCGCGTACTGAAACAGAAAAAGAAATTGCTGCTGTGTGGGAAAAAGCACTGCGTCGTGAAAACGCCTCTGTGCAAGATGACTTCTTTGAGAGCGGCGGGAACTCCCTGATTGCGGTTGGTCTGGTTCGTGAACTGAACGCGCGTTTAGGCGTCAGCTTACCGCTGCAGTCTGTACTGGAGTCCCCGACCATTGAAAAACTGGCACGTCGCCTGGAACGTGAAGTGGCGCAGGAATCAAGCCGTTTTGTTCGTTTACACGCAGAAACTGGTAAAGCGCGTCCGGTGATTTGCTGGCCGGGCCTGGGCGGTTATCCTATGAACCTGCGTAGCCTGGCAGGTGAAATCGGCCTGGGCCGTAGCTTCTATGGTGTGCAGAGCTACGGCATTAACGAAGGTGAAACCCCGTATGAAACTATCACCGAGATGGCGAAAAAAGATATCGAGGCTCTGAAAGAAATCCAGCCGGCGGGGCCGTATACCCTGTGGGGCTACTCTTTCGGTGCGCGCGTTGCGTTTGAAACCGCCTATCAGCTGGAACAGGCTGGTGAAAAAGTGGATAACCTGTTCCTGATTGCACCGGGCTCACCGAAAGTTCGCGCTGAGAACGGTAAAGTTTGGGGTCGCGAAGCGAGCTTCGCAAACCGCGGATACACGACCATCCTGTTTTCTGTGTTTACTGGCACCATTTCCGGCCCAGACCTGGACCGTTGCCTGGAAACCGTGACTGATGAAGCATCCTTCGCTGAGTTCATCTCAGAGCTGAAAGGTATTGACGTTGACCTGGCGCGTCGCATCATCAGCGTAGTGGGCCAGACGTACGAGTTCGAATACTCTTTCCATGAACTGGCGGAGCGTACCCTGCAGGCGCCGATCTCTATCTTCAAAGCCGTGGGCGATGACTACTCCTTCCTGGAAAATAGCTCTGGCTATTCCGCGGAACCGCCGACCGTGATCGATCTGGATGCGGATCATTACAGCCTGCTGCGTGAAGATATCGGTGAACTGGTAAAACATATCCGCTATCTCCTGGGTGAATAATTAAGGAGGTAAAAAAAATGCGTGCGATGAACGATCGTCTGCCGTCCTTCTGCACCCCGCTGGATGATCGTTGGCCGCTGCCGGTTGCGCTGCCGGGCGTGCAGCTGCGTTCGACCCGCTTCGATCCGGCGCTGCTGCAGCCGGGCGATTTCGCGCTGGCGGGCATCCAGCCGCCGGCGAACATCCTGCGTGCGGTGGCGAAACGTCAGGCGGAGTTCCTGGCGGGTCGTCTGTGCGCGCGTGCGGCGCTGTTCGCGCTGGATGGTCGTGCGCAGACTCCGGCTGTTGGCGAAGATCGTGCGCCGGTTTGGCCGGCGGCGATCTCCGGCTCTATCACCCACGGCGATCGCTGGGCGGCGGCGCTGGTTGCGGCGCGCGGCGATTGGCGTGGTCTGGGTCTGGATGTTGAAACCCTGCTGGAAGCGGAACGTGCGCGTTACCTGCACGGCGAAATCCTGACCGAAGGCGAACGTCTGCGTTTCGCGGATGATCTGGAACGTCGTACCGGCCTGCTGGTGACCCTGGCGTTCTCCCTGAAAGAAAGCCTGTTCAAAGCGCTGTACCCGCTGGTTGGCAAACGTTTCTACTTCGAACACGCAGAACTGCTGGAATGGCGTGCGGACGGTCAAGCGCGTCTGCGTCTGCTGACCGATCTGAGCCCGGAATGGCGTCACGGTTCTGAACTGGATGCGCAGTTCGCGGTTCTGGACGGCCGTCTGCTGTCTCTGGTTGCGGTTGGTGCGtaaGAGCTC | | *Bgl* II  *Sac* I |
| pnK12-ABS-ind | AGATCTCTGCACTTACACATTCGTTAAGTCATATATGTTTTTGACTTATCCGCTTCGAAGAGAGACACTACCTGCAACAATCAGGAGCGCAATATGTCATTTCTGTTACCCATCCAATTGTTCAAAATTCTTGCTGATGAAACCCGTCTGGGCATCGTTTTACTGCTCAGCGAACTGGGAGAGTTATGCGTCTGCGATCTCTGCACTGCTCTCGACCAGTCGCAGCCCAAGATCTCCCGCCACCTGGCATTGCTGCGTGAAAGCGGGCTATTGCTGGACCGCAAGCAAGGTAAGTGGGTTCATTACCGCTTATCACCGCATATTCCAGCATGGGCGGCGAAAATTATTGATGAGGCCTGGCGATGTGAACAGGAAAAGGTTCAGGCGATTGTCCGCAACCTGGCTCGACAAAACTGTTCCGGGGACAGTAAGAACATTTGCAGTTAATACACATTCGTTAAGTCATATATGTTTTTGACTTATCTAGAAATAATTTTGTTTAACTTTAAGAAGGAGATATACATATGACCCTGCAGGAAACCTCTGTTCTGGAACCGACCCTGCAGGGCACCACCACGCTGCCGGGTCTGCTGGCTCAGCGCGTAGCTGAACACCCGGAAGCTATCGCAGTTGCTTACCGTGATGACAAACTGACCTTCCGTGAACTGGCTTCTCGCTCGGCCGCGCTGGCGGACTACCTGGAACACCTGGGTGTCTCCGCGGACGACTGTGTTGGTCTGTTCGTAGAACCGTCTATCGACCTGATGGTTGGCGCGTGGGGCATCCTGAACGCCGGCGCGGCATACCTGCCGCTGAGCCCGGAATACCCGGAAGATCGCCTGCGTTACATGATCGAAAACAGCGAAACTAAAATTATCCTGGCCCAGCAGCGCCTGGTTAGCCGCCTGCGTGAACTCGCCCCGAAAGACGTTACCATTGTTACTTTACGTGAATCTGAAGCCTTTGTGCGTCCGGAAGGCACGGAAGCGCCGGCGGCACGCTCCGCTCGTCCGGACACCCTGGCCTACGTGATCTATACTTCCGGTAGCACCGGAAAACCGAAAGGTGTTATGATTGAACACCGCTCTATCGTTAACCAACTGGGCTGGCTGCGTGAAACCTACGCCATCGACCGTTCTAAAGTAATTCTGCAGAAAACTCCGATGTCGTTCGACGCTGCTCAGTGGGAAATTCTGTCTCCGGCTAACGGCGCGACCGTTGTTATGGGCGCACCGGGTGTCTACGCGGACCCGGAAGGTCTGATCGAAACCATCGTGAAACATAACGTTACCACTCTGCAGTGCGTTCCGACCCTGCTGCAGGGTCTGATTGATACCGAGAAATTCCCGGAATGCGTAAGTCTGCAGCAGATTTTCTCCGGCGGTGAAGCGCTGTCCCGCCTGCTGGCCATCCAGACCACTCAAGAAATGCCGGGCCGTGCCCTGATCAACGTTTACGGCCCTACAGAAACCACGATTAACAGCTCCTCTTTTCCGGTAGATCCGGCAGACCTCGATGAAGGCCCGCAGAGCATCAGCATCGGTAGCCCGGTACACGGTACCACCTACCACATCCTGGACAAAGAAACCCTCAAACCGGTCGGCGTCGGCGAAATTGGTGAACTGTATATCGGCGGCATTCAGCTGGCTCGCGGCTACCTGCACCGTGACGACCTGACTGCCGAACGCTTCCTGGAGATTGAACTGGAAGAAGGTGCGGAACCGGTTCGCTTGTATAAAACTGGTGATCTGGGTCAGTGGAACAACGATGGCACGGTTCAGTTCGCTGGTCGTGCAGACAACCAAGTGAAACTGCGTGGTTATCGCGTTGAACTTGATGAAATTAGCCTGGCGATTGAAAATCACGATTGGGTTCGTAACGCTGCAGTGATCGTGAAAAATGACGGTCGTACTGGTTTTCAGAACCTGATCGCTTGTATTGAACTGAGTGAAAAAGAAGCAGCACTGATGGATCAGGGTAATCATGGTTCACATCATGCTTCTAAAAAATCCAAACTGCAGGTGAAGGCCCAGCTGAGCAATCCGGGTCTGCGTGATGATGCGGAATTAGCCGCTCGCCCGGCCTTCGACCTGGAAGGTGCAGAGCCAACCCCTGAACAACGTGCGCGTGTTTTTGCGCGCAAAACGTACCGTTTCTACGAGGGCGGCGCGGTGACGCAGGCTGACTTGCTGGGCCTGCTGGGTGCGACCGTGACCGCGGGTTATTCTCGTAAAGCTGCCGATCTGGCACCGGCGGAACTGGGCCAGATTCTGCGTTGGTTCGGCCAGTACATTTCTGAAGAACGTCTGTTACCAAAATATGGTTACGCTTCTCCTGGTGCTCTGTACGCAACCCAAATGTACTTCGAACTGGAAGGTGTTGGCGGTTTGAAACCGGGTTATTATTACTACCAACCGGTTCGTCACCAGCTCGTGTTGATTTCTGAACGTGAAGCCACCGGTAAAGCCACCGCACAGATCCACTTCATTGGCAAAAAGAGTGGCATTGAACCGGTTTATAAAAATAACATCTTGGAAGTTCTTGAAATTGAAACTGGTCACATGGTTGGTCTGTTCGAACAGATCCTGCCGGCATACGGCCTCGATATTCATGATCGTGCGTACGAACCGGCTGTTAAGGATCTGCTGGACGTTGCGGATGAGGACTACTACCTGGGCACTTTCGAACTGGTTCCGCACGCGGGTGCACGCGACGATCAGGCGGAAGTCTATGTACAGACCCACGGTGGCAAAGTAGCTGGTCTGCCGGAAGGTCAGTATCGCTATGAAAATGGTGAGTTGACTCGCTTCAGCGACGACATTGTTCTGAAAAAACACGTTATTGCTATCAATCAGTCCGTCTACCAAGCTGCGTCCTTTGGTATCAGCGTTTACTCGCGTGCAGAAGAAGAATGGTTAAAGTACATCACGCTGGGTAAAAAACTGCAACATCTGATGATGAACGGTCTTAACCTGGGCTTTATGTCCAGCGGTTACTCCTCTAAAACCGGTAATCCGCTGCCGGCGTCCCGCCGTATGGATGCTGTTCTGGGTGCTAACGGCGTGGACAGCGCGCCAATGTATTTCTTTGTTGGTGGCCGCATTTCGGACGAACAGATCGGTCACGAAGGTATGCGTGAAGATTCCGTGCACATGCGCGGTCCGGCTGAACTGATCCGTGACGACCTGGTTAGCTTCCTGCCGGACTATATGATCCCGAACCGTGTCGTGGTATTCGATCGTCTGCCACTGAGCGCGAACGGTAAAATCGACGTTAAAGCACTGGCGGCCTCTGATCAGGTGAACGCGGAGCTGGTTGAACGTCCGTTTGTCGCACCGCGTACTGAAACAGAAAAAGAAATTGCTGCTGTGTGGGAAAAAGCACTGCGTCGTGAAAACGCCTCTGTGCAAGATGACTTCTTTGAGAGCGGCGGGAACTCCCTGATTGCGGTTGGTCTGGTTCGTGAACTGAACGCGCGTTTAGGCGTCAGCTTACCGCTGCAGTCTGTACTGGAGTCCCCGACCATTGAAAAACTGGCACGTCGCCTGGAACGTGAAGTGGCGCAGGAATCAAGCCGTTTTGTTCGTTTACACGCAGAAACTGGTAAAGCGCGTCCGGTGATTTGCTGGCCGGGCCTGGGCGGTTATCCTATGAACCTGCGTAGCCTGGCAGGTGAAATCGGCCTGGGCCGTAGCTTCTATGGTGTGCAGAGCTACGGCATTAACGAAGGTGAAACCCCGTATGAAACTATCACCGAGATGGCGAAAAAAGATATCGAGGCTCTGAAAGAAATCCAGCCGGCGGGGCCGTATACCCTGTGGGGCTACTCTTTCGGTGCGCGCGTTGCGTTTGAAACCGCCTATCAGCTGGAACAGGCTGGTGAAAAAGTGGATAACCTGTTCCTGATTGCACCGGGCTCACCGAAAGTTCGCGCTGAGAACGGTAAAGTTTGGGGTCGCGAAGCGAGCTTCGCAAACCGCGGATACACGACCATCCTGTTTTCTGTGTTTACTGGCACCATTTCCGGCCCAGACCTGGACCGTTGCCTGGAAACCGTGACTGATGAAGCATCCTTCGCTGAGTTCATCTCAGAGCTGAAAGGTATTGACGTTGACCTGGCGCGTCGCATCATCAGCGTAGTGGGCCAGACGTACGAGTTCGAATACTCTTTCCATGAACTGGCGGAGCGTACCCTGCAGGCGCCGATCTCTATCTTCAAAGCCGTGGGCGATGACTACTCCTTCCTGGAAAATAGCTCTGGCTATTCCGCGGAACCGCCGACCGTGATCGATCTGGATGCGGATCATTACAGCCTGCTGCGTGAAGATATCGGTGAACTGGTAAAACATATCCGCTATCTCCTGGGTGAATAATTAAGGAGGTAAAAAAAATGCGTGCGATGAACGATCGTCTGCCGTCCTTCTGCACCCCGCTGGATGATCGTTGGCCGCTGCCGGTTGCGCTGCCGGGCGTGCAGCTGCGTTCGACCCGCTTCGATCCGGCGCTGCTGCAGCCGGGCGATTTCGCGCTGGCGGGCATCCAGCCGCCGGCGAACATCCTGCGTGCGGTGGCGAAACGTCAGGCGGAGTTCCTGGCGGGTCGTCTGTGCGCGCGTGCGGCGCTGTTCGCGCTGGATGGTCGTGCGCAGACTCCGGCTGTTGGCGAAGATCGTGCGCCGGTTTGGCCGGCGGCGATCTCCGGCTCTATCACCCACGGCGATCGCTGGGCGGCGGCGCTGGTTGCGGCGCGCGGCGATTGGCGTGGTCTGGGTCTGGATGTTGAAACCCTGCTGGAAGCGGAACGTGCGCGTTACCTGCACGGCGAAATCCTGACCGAAGGCGAACGTCTGCGTTTCGCGGATGATCTGGAACGTCGTACCGGCCTGCTGGTGACCCTGGCGTTCTCCCTGAAAGAAAGCCTGTTCAAAGCGCTGTACCCGCTGGTTGGCAAACGTTTCTACTTCGAACACGCAGAACTGCTGGAATGGCGTGCGGACGGTCAAGCGCGTCTGCGTCTGCTGACCGATCTGAGCCCGGAATGGCGTCACGGTTCTGAACTGGATGCGCAGTTCGCGGTTCTGGACGGCCGTCTGCTGTCTCTGGTTGCGGTTGGTGCGtaaGAGCTC | | *Bgl* II  *Sac* I |
| pnK12-ABS-ind-GlpF | AGATCTCTGCACTTACACATTCGTTAAGTCATATATGTTTTTGACTTATCCGCTTCGAAGAGAGACACTACCTGCAACAATCAGGAGCGCAATATGTCATTTCTGTTACCCATCCAATTGTTCAAAATTCTTGCTGATGAAACCCGTCTGGGCATCGTTTTACTGCTCAGCGAACTGGGAGAGTTATGCGTCTGCGATCTCTGCACTGCTCTCGACCAGTCGCAGCCCAAGATCTCCCGCCACCTGGCATTGCTGCGTGAAAGCGGGCTATTGCTGGACCGCAAGCAAGGTAAGTGGGTTCATTACCGCTTATCACCGCATATTCCAGCATGGGCGGCGAAAATTATTGATGAGGCCTGGCGATGTGAACAGGAAAAGGTTCAGGCGATTGTCCGCAACCTGGCTCGACAAAACTGTTCCGGGGACAGTAAGAACATTTGCAGTTAATACACATTCGTTAAGTCATATATGTTTTTGACTTATCTAGAAATAATTTTGTTTAACTTTAAGAAGGAGATATACATATGACCCTGCAGGAAACCTCTGTTCTGGAACCGACCCTGCAGGGCACCACCACGCTGCCGGGTCTGCTGGCTCAGCGCGTAGCTGAACACCCGGAAGCTATCGCAGTTGCTTACCGTGATGACAAACTGACCTTCCGTGAACTGGCTTCTCGCTCGGCCGCGCTGGCGGACTACCTGGAACACCTGGGTGTCTCCGCGGACGACTGTGTTGGTCTGTTCGTAGAACCGTCTATCGACCTGATGGTTGGCGCGTGGGGCATCCTGAACGCCGGCGCGGCATACCTGCCGCTGAGCCCGGAATACCCGGAAGATCGCCTGCGTTACATGATCGAAAACAGCGAAACTAAAATTATCCTGGCCCAGCAGCGCCTGGTTAGCCGCCTGCGTGAACTCGCCCCGAAAGACGTTACCATTGTTACTTTACGTGAATCTGAAGCCTTTGTGCGTCCGGAAGGCACGGAAGCGCCGGCGGCACGCTCCGCTCGTCCGGACACCCTGGCCTACGTGATCTATACTTCCGGTAGCACCGGAAAACCGAAAGGTGTTATGATTGAACACCGCTCTATCGTTAACCAACTGGGCTGGCTGCGTGAAACCTACGCCATCGACCGTTCTAAAGTAATTCTGCAGAAAACTCCGATGTCGTTCGACGCTGCTCAGTGGGAAATTCTGTCTCCGGCTAACGGCGCGACCGTTGTTATGGGCGCACCGGGTGTCTACGCGGACCCGGAAGGTCTGATCGAAACCATCGTGAAACATAACGTTACCACTCTGCAGTGCGTTCCGACCCTGCTGCAGGGTCTGATTGATACCGAGAAATTCCCGGAATGCGTAAGTCTGCAGCAGATTTTCTCCGGCGGTGAAGCGCTGTCCCGCCTGCTGGCCATCCAGACCACTCAAGAAATGCCGGGCCGTGCCCTGATCAACGTTTACGGCCCTACAGAAACCACGATTAACAGCTCCTCTTTTCCGGTAGATCCGGCAGACCTCGATGAAGGCCCGCAGAGCATCAGCATCGGTAGCCCGGTACACGGTACCACCTACCACATCCTGGACAAAGAAACCCTCAAACCGGTCGGCGTCGGCGAAATTGGTGAACTGTATATCGGCGGCATTCAGCTGGCTCGCGGCTACCTGCACCGTGACGACCTGACTGCCGAACGCTTCCTGGAGATTGAACTGGAAGAAGGTGCGGAACCGGTTCGCTTGTATAAAACTGGTGATCTGGGTCAGTGGAACAACGATGGCACGGTTCAGTTCGCTGGTCGTGCAGACAACCAAGTGAAACTGCGTGGTTATCGCGTTGAACTTGATGAAATTAGCCTGGCGATTGAAAATCACGATTGGGTTCGTAACGCTGCAGTGATCGTGAAAAATGACGGTCGTACTGGTTTTCAGAACCTGATCGCTTGTATTGAACTGAGTGAAAAAGAAGCAGCACTGATGGATCAGGGTAATCATGGTTCACATCATGCTTCTAAAAAATCCAAACTGCAGGTGAAGGCCCAGCTGAGCAATCCGGGTCTGCGTGATGATGCGGAATTAGCCGCTCGCCCGGCCTTCGACCTGGAAGGTGCAGAGCCAACCCCTGAACAACGTGCGCGTGTTTTTGCGCGCAAAACGTACCGTTTCTACGAGGGCGGCGCGGTGACGCAGGCTGACTTGCTGGGCCTGCTGGGTGCGACCGTGACCGCGGGTTATTCTCGTAAAGCTGCCGATCTGGCACCGGCGGAACTGGGCCAGATTCTGCGTTGGTTCGGCCAGTACATTTCTGAAGAACGTCTGTTACCAAAATATGGTTACGCTTCTCCTGGTGCTCTGTACGCAACCCAAATGTACTTCGAACTGGAAGGTGTTGGCGGTTTGAAACCGGGTTATTATTACTACCAACCGGTTCGTCACCAGCTCGTGTTGATTTCTGAACGTGAAGCCACCGGTAAAGCCACCGCACAGATCCACTTCATTGGCAAAAAGAGTGGCATTGAACCGGTTTATAAAAATAACATCTTGGAAGTTCTTGAAATTGAAACTGGTCACATGGTTGGTCTGTTCGAACAGATCCTGCCGGCATACGGCCTCGATATTCATGATCGTGCGTACGAACCGGCTGTTAAGGATCTGCTGGACGTTGCGGATGAGGACTACTACCTGGGCACTTTCGAACTGGTTCCGCACGCGGGTGCACGCGACGATCAGGCGGAAGTCTATGTACAGACCCACGGTGGCAAAGTAGCTGGTCTGCCGGAAGGTCAGTATCGCTATGAAAATGGTGAGTTGACTCGCTTCAGCGACGACATTGTTCTGAAAAAACACGTTATTGCTATCAATCAGTCCGTCTACCAAGCTGCGTCCTTTGGTATCAGCGTTTACTCGCGTGCAGAAGAAGAATGGTTAAAGTACATCACGCTGGGTAAAAAACTGCAACATCTGATGATGAACGGTCTTAACCTGGGCTTTATGTCCAGCGGTTACTCCTCTAAAACCGGTAATCCGCTGCCGGCGTCCCGCCGTATGGATGCTGTTCTGGGTGCTAACGGCGTGGACAGCGCGCCAATGTATTTCTTTGTTGGTGGCCGCATTTCGGACGAACAGATCGGTCACGAAGGTATGCGTGAAGATTCCGTGCACATGCGCGGTCCGGCTGAACTGATCCGTGACGACCTGGTTAGCTTCCTGCCGGACTATATGATCCCGAACCGTGTCGTGGTATTCGATCGTCTGCCACTGAGCGCGAACGGTAAAATCGACGTTAAAGCACTGGCGGCCTCTGATCAGGTGAACGCGGAGCTGGTTGAACGTCCGTTTGTCGCACCGCGTACTGAAACAGAAAAAGAAATTGCTGCTGTGTGGGAAAAAGCACTGCGTCGTGAAAACGCCTCTGTGCAAGATGACTTCTTTGAGAGCGGCGGGAACTCCCTGATTGCGGTTGGTCTGGTTCGTGAACTGAACGCGCGTTTAGGCGTCAGCTTACCGCTGCAGTCTGTACTGGAGTCCCCGACCATTGAAAAACTGGCACGTCGCCTGGAACGTGAAGTGGCGCAGGAATCAAGCCGTTTTGTTCGTTTACACGCAGAAACTGGTAAAGCGCGTCCGGTGATTTGCTGGCCGGGCCTGGGCGGTTATCCTATGAACCTGCGTAGCCTGGCAGGTGAAATCGGCCTGGGCCGTAGCTTCTATGGTGTGCAGAGCTACGGCATTAACGAAGGTGAAACCCCGTATGAAACTATCACCGAGATGGCGAAAAAAGATATCGAGGCTCTGAAAGAAATCCAGCCGGCGGGGCCGTATACCCTGTGGGGCTACTCTTTCGGTGCGCGCGTTGCGTTTGAAACCGCCTATCAGCTGGAACAGGCTGGTGAAAAAGTGGATAACCTGTTCCTGATTGCACCGGGCTCACCGAAAGTTCGCGCTGAGAACGGTAAAGTTTGGGGTCGCGAAGCGAGCTTCGCAAACCGCGGATACACGACCATCCTGTTTTCTGTGTTTACTGGCACCATTTCCGGCCCAGACCTGGACCGTTGCCTGGAAACCGTGACTGATGAAGCATCCTTCGCTGAGTTCATCTCAGAGCTGAAAGGTATTGACGTTGACCTGGCGCGTCGCATCATCAGCGTAGTGGGCCAGACGTACGAGTTCGAATACTCTTTCCATGAACTGGCGGAGCGTACCCTGCAGGCGCCGATCTCTATCTTCAAAGCCGTGGGCGATGACTACTCCTTCCTGGAAAATAGCTCTGGCTATTCCGCGGAACCGCCGACCGTGATCGATCTGGATGCGGATCATTACAGCCTGCTGCGTGAAGATATCGGTGAACTGGTAAAACATATCCGCTATCTCCTGGGTGAATAATTAAGGAGGTAAAAAAAATGCGTGCGATGAACGATCGTCTGCCGTCCTTCTGCACCCCGCTGGATGATCGTTGGCCGCTGCCGGTTGCGCTGCCGGGCGTGCAGCTGCGTTCGACCCGCTTCGATCCGGCGCTGCTGCAGCCGGGCGATTTCGCGCTGGCGGGCATCCAGCCGCCGGCGAACATCCTGCGTGCGGTGGCGAAACGTCAGGCGGAGTTCCTGGCGGGTCGTCTGTGCGCGCGTGCGGCGCTGTTCGCGCTGGATGGTCGTGCGCAGACTCCGGCTGTTGGCGAAGATCGTGCGCCGGTTTGGCCGGCGGCGATCTCCGGCTCTATCACCCACGGCGATCGCTGGGCGGCGGCGCTGGTTGCGGCGCGCGGCGATTGGCGTGGTCTGGGTCTGGATGTTGAAACCCTGCTGGAAGCGGAACGTGCGCGTTACCTGCACGGCGAAATCCTGACCGAAGGCGAACGTCTGCGTTTCGCGGATGATCTGGAACGTCGTACCGGCCTGCTGGTGACCCTGGCGTTCTCCCTGAAAGAAAGCCTGTTCAAAGCGCTGTACCCGCTGGTTGGCAAACGTTTCTACTTCGAACACGCAGAACTGCTGGAATGGCGTGCGGACGGTCAAGCGCGTCTGCGTCTGCTGACCGATCTGAGCCCGGAATGGCGTCACGGTTCTGAACTGGATGCGCAGTTCGCGGTTCTGGACGGCCGTCTGCTGTCTCTGGTTGCGGTTGGTGCGtaaGAGCTCTCGAGATTACACTTTATGCTTCCGGCTCGTATAATGTGTGGattaaagaggagaaaATGAGCCAGACTTCCACTCTGAAAGGTCAATGTATTGCTGAATTTCTGGGCACCGGTCTGCTTATTTTCTTCGGCGTTGGCTGTGTTGCGGCGCTGAAAGTTGCGGGCGCGTCTTTCGGACAGTGGGAAATCAGCGTTATCTGGGGTCTGGGCGTTGCGATGGCGATCTACCTGACCGCGGGCGTTTCTGGTGCGCACCTGAACCCGGCGGTTACCATCGCGCTGTGGCTGTTCGCGTGCTTCGATAAACGTAAAGTTATCCCGTTCATCGTTTCTCAGGTTGCGGGTGCTTTCTGCGCGGCGGCGCTGGTTTACGGCCTGTACTACAACCTGTTCTTCGATTTCGAACAGACCCACCACATCGTTCGTGGTAGCGTTGAATCCGTTGATCTGGCGGGCACCTTCAGCACCTACCCGAACCCGCACATCAACTTCGTTCAGGCGTTCGCGGTTGAAATGGTTATCACCGCGATCCTGATGGGCCTGATCCTGGCGCTGACCGATGACGGCAACGGTGTTCCGCGTGGCCCGCTGGCGCCGCTGCTGATCGGCCTGCTGATCGCGGTTATCGGTGCATCTATGGGTCCGCTGACCGGTTTCGCGATGAACCCGGCGCGTGATTTCGGCCCGAAAGTTTTCGCGTGGCTGGCGGGTTGGGGTAACGTTGCGTTCACCGGCGGTCGTGATATTCCGTATTTCTTAGTTCCGCTGTTCGGCCCGATTGTTGGCGCTATCGTTGGTGCATTCGCTTATCGTAAACTGATCGGCCGTCACCTGCCGTGCGATATCTGCGTTGTTGAAGAAAAAGAAACCACCACCCCGTCTGAACAGAAAGCGAGCCTGtaaAAGCTT | | *Bgl* II  *Hin*d III |

The constitutive promoter P*_cueR_* and P_637_ is shown in a turquoise background. The constitutive promoter P_J23119_ is shown on a yellow background. The arsenic sensory promoter is in a grey background; the ArsR-binding site is in a dark gray background, and the ArsR encoding sequence in blue. The start codon is shown on a green background. The end codon is shown on a red background. The ribosome binding site is shown in orange. The *bps*A-*pcp*S gene cluster is shown in dark blue. The mCherry encoding sequence is shown in red. The GlpF encoding sequence is shown in dark yellow, and the GlpF encoding sequence is shown in cyan. Restriction sites are underlined.


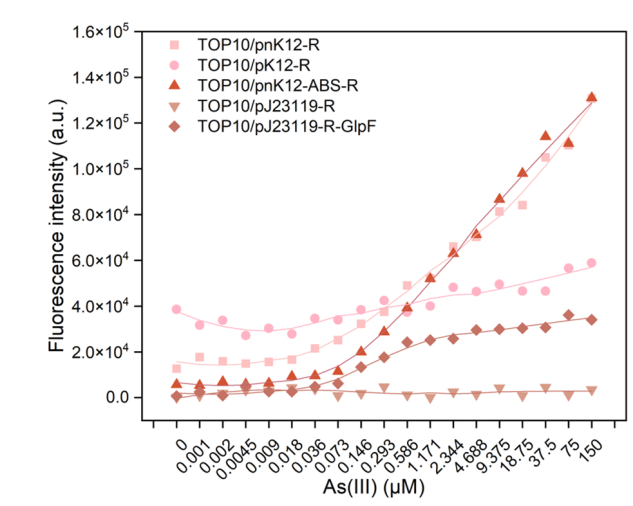


**FIG S1 Dose-response profiles of red fluorescent protein-based biosensors for arsenic detection.** This figure encapsulates the dose-response curves under conditions of As(III) induction ranging from 0 to 150 μM at a controlled temperature of 37°C and agitation speed of 250 rpm for 4 hours. Analyzing the dose-response relationship between As(III) concentration and the corresponding fluorescence intensity provides insights into these biosensors' sensitivity and dynamic range.
